# Genetic Characterization of a Novel *Equus caballus* Papillomavirus Isolated from a Thoroughbred Mare

**DOI:** 10.3390/v15030650

**Published:** 2023-02-28

**Authors:** Silvia Turco, Federica Gabbianelli, Carla N. Mavian, Daniele Pietrucci, Livia De Paolis, Rodolfo Gialletti, Luca Mechelli, Chiara Grazia De Ciucis, Katia Cappelli, Filippo Dell’Anno, Samanta Mecocci, Gian Guido Donato, Tiziana Nervo, Floriana Fruscione, Maria Ines Crescio, Alessandro Ghelardi, Giovanni Chillemi, Elisabetta Razzuoli

**Affiliations:** 1 Dipartimento di Scienze Agrarie e Forestali (DAFNE), Università degli Studi della Tuscia, Via San Camillo de Lellis snc, 01100 Viterbo, Italy; 2 Dipartimento per la Innovazione nei Sistemi Biologici, Agroalimentari e Forestali (DIBAF), Università degli Studi della Tuscia, Via San Camillo de Lellis snc, 01100 Viterbo, Italy; 3 Emerging Pathogen Institute, University of Florida, Gainesville, FL 32608, USA; 4 Department of Pathology, Immunology and Laboratory Medicine, University of Florida, Gainesville, FL 32610, USA; 5 Istituto Zooprofilattico Sperimentale del Piemonte, Liguria e Valle d’Aosta, National Reference Center of Veterinary and comparative Oncology (CEROVEC), Piazza Borgo Pila 29/34, 16129 Genova, Italy; 6 Dipartimento di Medicina Veterinaria, Università degli Studi di Perugia, Via San Costanzo 4, 06126 Perugia, Italy; 7 Dipartimento di Scienze Veterinarie, Università degli Studi di Torino, Largo Paolo Braccini 2, 10095 Grugliasco (TO), Italy; 8 Azienda Usl Toscana Nord-Ovest, UOC Ostetricia e Ginecologia, Nuovo Ospedale Apuane, Via Enrico Mattei 21, 54100 Massa, Italy

**Keywords:** EcPVs, virus detection, Italy, horse, EcPV10

## Abstract

Papillomaviruses (PVs) are small, non-enveloped viruses, ubiquitous across the animal kingdom. PVs induce diverse forms of infection, such as cutaneous papillomas, genital papillomatosis, and carcinomas. During a survey on the fertility status of a mare, a novel *Equus caballus* PV (EcPV) has been identified using Next Generation Sequencing, and it was further confirmed with genome-walking PCR and Sanger sequencing. The complete circular genome 7607 bp long shares 67% average percentage of identity with EcPV9, EcPV2, EcPV1, and EcPV6, justifying a new classification as *Equus caballus* PV 10 (EcPV10). All EcPV genes are conserved in EcPV10, and phylogenetic analysis indicates that EcPV10 is closely related to EcPV9 and EcPV2, genus Dyoiota 1. A preliminary EcPV10 genoprevalence study, carried out on 216 horses using Real Time PCRs, suggested a low incidence of this isolate (3.7%) compared to EcPVs of the same genus such as EcPV2 and EcPV9 in the same horse population. We hypothesize a transmission mechanism different from the one observed in the closely related EcPV9 and EcPV2 that particularly infect Thoroughbreds. This horse breed is usually submitted to natural mating, thus indicating a possible sexual diffusion. No differences were detected for breeds in terms of susceptibility to EcPV10. Further studies are needed to investigate the molecular mechanisms behind the host and EcPV10 infection to explain the reduced viral spread.

## 1. Introduction

Papillomaviruses (PVs) are small, non-enveloped DNA viruses, able to infect a wide variety of vertebrates. More than 240 types of PVs have been described in mammals [1]. In humans, PV infections are mostly asymptomatic and are mainly resolved by the host’s immune response. This tolerated partnership allows the virus to replicate and persist in the host, without the pressure of evolutionary events [2]. On the contrary, unsolved chronic high-risk PV infections can lead to uncontrolled cell proliferation and neoplastic transformations [2]. Upon infection, the viral genome can be either integrated into the host DNA or maintained as multiple episomes replicate concomitantly with the host cells. Usually, the integration is correlated with higher disease severity [3,4].

To date, 14 species of PVs have been reported to infect equines such as horses and donkeys: three bovine PVs (*Bos taurus* papillomavirus 1 (BPV1), 2 (BPV2), and 13 (BPV13)), two *Equus asinus* PVs 1–2 (EaPV1 and EaPV2), and nine *Equus caballus* PVs 1–9 (EcPV1–9) [5]. As it occurs in humans, PV infections in horses can lead to the development or progression of different kinds of lesions, such as aural plaques, genital masses, and verrucous lesions [5]. In particular, EcPV1 and EcPV8 have been associated with cutaneous papillomas and papillomatosis [6,7]; EcPV2 and EcPV3 with penile and preputial squamous cell carcinoma (SCC) [8]; EcPV1, 3, 4, 5, 6 and 7 with aural and genital plaques, with a high percentage of co-infection as well [9,10]. Furthermore, EcPV2 has been reported in penile, vulvar, clitoral, and oropharyngeal papillomas, as well as in both in situ carcinoma (CIS) and invasive SCCs [11,12,13]. PVs’ genome sizes range from 6953 bp (*Chelonia mydas* papillomavirus type 1, CmPV1) to 8607 bp (*Canine* papillomavirus type 1, CPV1) [1]. Regardless of host species and PV type, all the PV genomes encode for at least five genes, divided into early (E) and late (L) genes according to their expression during the infection cycle, and an upstream regulatory region (URR) contains enhancers and promoters [3]. E1 is a helicase involved in replication, while E2 is a transcription factor that enhances E1 and E2 expression, both expressed during the early stage of the infection [14]. E4 is contained within E2 Open Reading Frame (ORF), and it is actively spliced to finalize virus amplification and synthesis [15]. L1 and L2, instead, encode for the icosahedral capsid protein and for a structural protein that helps the packing into viral particles, both expressed during the late infection. Three additional genes (E5, E6, E7), involved in cell growth and immune response, are considered accessory genes acquired during the PV evolution, and their presence vary among the different phylogenetic groups [1]. Among the PV genes, L1 is the most conserved one and, for this reason, used for PV detection and identification [3,16]. Hence, according to their relative clinical history and conditions, horses admitted to the veterinary hospital are routinely tested for the presence of PVs using specific detection and quantification methods targeting the L1 gene.

In this study, we report the identification of a new EcPV type named EcPV10, isolated from a vaginal cytobrush of an infertile mare, and provide its completely assembled genome. This sequence has been compared to closely related PVs, and the genoprevalence of this virus has been tested in 216 samples obtained from horse genital tracts.

## 2. Materials and Methods

### 2.1. Anamnesis and Sampling

A Thoroughbred of seven years old from Chillivani (Sardinia, Italy) was admitted in February 2021 to the Veterinary Teaching Hospital (OVU) of the University of Turin (Italy) for subfertility. During the previous breeding season, she was subjected to natural mating four times with a proven fertility stallion but failed to become pregnant. However, clinical signs of endometritis were not observed during any cycle (excessive intrauterine fluid accumulation before and/or after breeding, vaginal discharge, short inter-oestrous intervals). Therefore, after the admission, the mare was submitted to a complete reproductive tract examination, including transrectal palpation, ultrasound examination (MyLabOne, Esaote, Genova, Italy), and vaginal speculum examination. Furthermore, all the procedures routinely used to diagnose uterine illnesses in the mare were performed [17], including cytological examination [18], endometrial culture and histological examination [19], and PCR for *Chlamydia* spp. detection [20]. None of these procedures were able to identify a clear and convincing cause of infertility.

In light of these results, screening for EcPV2 infection was performed, following the protocol described in Cappelli et al. (2022) [13]. Cytobrush (Deltalab SLU, Barcelona, Spain) sampling was carried out through mild rubbing on the vulvar mucosa. The brush was moisturized in 800 µL of DNA/RNA Shield Stabilization Solution (Zymo Research, Irvine, CA, USA) and stored at −20 °C until extractions.

### 2.2. DNA Sequencing and De Novo Assembly

Total DNA was extracted from three biological replicates using a QIAamp DNA Mini Kit (QIAGEN, Milano, Italy), according to manufacturer’s instructions, and sequenced independently with an Illumina NovaSeq 6000 machine using paired-end sequencing methods at the Institute of Applied Genomics (IGA, Udine, Italy). Raw reads quality was evaluated with FastQC [21], and the Illumina adapters were removed using Trimmomatic v0.39 [22] using the following parameters: LEADING:15, TRAILING:15, SLIDINGWINDOW:4:15, MINLEN:111, HEADCROP:11. Trimmed viral reads were filtered using the host *Equus caballus* genome (EquCab2.0, GCF_000002305.2) as reference with BWA v 0.7.12 aligner and samtools v1.13 [23,24]. De novo assembly of the filtered reads was performed using SPAdes v3.15.4 [25] with default parameters and k-mers set to 21, 33, 55, and 77.

To check the presence of any possible EcPV, assembled contigs were compared using BLASTn algorithm against an in-house built database that included the EcPV genomes available on NCBI database at the time of this analysis (Table S1). Contigs that were related, even if distantly, to EcPV were aligned to each other for genome reconstruction. The sequence was further validated with filtered-raw-reads alignment using BWA [23]. The alignment was visualized through the Integrative Genomics Viewer [26], and breadth and depth coverage were evaluated with Qualimap [27].

### 2.3. Genome Annotation and Phylogenetic Analysis

Viral ORFs were identified through the NCBI ORF Finder, Gatu [28], and PuMA [29]. A schematic genome representation was carried out through Cgview [30]. To perform phylogenetic analysis, the sequence of the E1, E2, L1, and L2 genes was retrieved from 55 bovine and equine papillomavirus species listed in Table S1 and aligned with Virulign [31] using default parameters. A Maximum Likelihood (ML) tree was built with RaxML-HPC using the GTRCATI algorithm as nucleotide substitution model and 1000 bootstrap [32]. The ML tree was visualized with FigTree v.1.4.4 http://tree.bio.ed.ac.uk/software/figtree (accessed on 21 September 2022) and edited with Inkscape v0.92 www.inkscape.org (accessed on 22 September 2022). The amino acid (aa) sequences were aligned with BLASTp to investigate similarity with closely related oncoproteins.

### 2.4. PCR Amplification and Sanger Sequencing

A genome walking approach was developed by designing overlapping PCR primers, listed in Table 1. Touchdown PCR was performed as follows: 2.5 μL of DNA (10 ng) were incubated with 0.4 μM of both forward and reverse primer, 1× of BiolineTM (Meridien Bioscience) reaction mix containing ultra-stableTaq DNA polymerase in a final volume of 25 μL. PCRs were performed with MJ Research PTC-200 Thermal Cycler using the following program: 5 min denaturation step at 95 °C, followed by 15 cycles of denaturation at 95 °C for 30 s, annealing starting from 62 °C and decreasing 0.5 °C per cycle for 30 s, extension at 72 °C for 30 s, followed by further 15 cycles of denaturation at 95 °C for 30 s, annealing at 58 °C for 30 s, and extension at 72 °C for 30 s, with a final elongation step carried out at 72 °C for 10 min. PCR products were visualized and evaluated on a 2.2% FlashGel (Lonza) and purified with ExoSAP-IT (ThermoFisher Scientific, Waltham, MA, USA), according to the manufacturer’s instructions. PCR amplicons were Sanger sequenced at Eurofins genomics (Eurofins Genomics GmbH, Konstanz, Germany) and aligned to the assembled sequence with BLASTn.

### 2.5. Development of a Real Time Pcr-Based Specific Assay

To develop a Real Time PCR assay specific to the newly assembled EcPV genome, primers and a probe targeting the L1 gene were designed (Table 1). Their specificity was tested in silico by using the in-house built EcPV genome database as a custom-defined database in the Primer-BLAST tool, as well as by blasting the resulting amplicon towards the *Papillomaviridae* (taxid:151340) NCBI database. qPCR reactions were prepared with 1× iTAQ universal probes supermix (BioRad, Milan, Italy), 1 μM of both forward and reverse primers, 0.2 μM of the probe, and 2 μL of DNA, in a final volume of 25 μL of double sterile water. Thermal cycling parameters consisted of an initial preheating step for 5 min at 95 °C followed by 40 cycles at 95 °C for 15 s, 60 °C for 45 s, performed in a Rotor-gene Q machine (QIAGEN). The acquisition of the fluorescent signal occurred at the end of each cycle, and the threshold to consider the sample positive was set at cycle 38.

### 2.6. Genital Brush Samples Collection from Horses for EcPV10 Detection

From January 2021 to July 2022, genital brushes were collected at the Didactic Veterinary University Hospital (OVUD) of Perugia and Turin for reproductive reasons, with the following inclusion criteria: (i) no sign of neoplastic disease, (ii) no lesions were associated to PVs infection. Penile and vulvar swabs were collected through sampling with cytobrushes (Deltalab SLU, Barcelona, Spain) as previously described [13]. Brushes were stored in 2 mL tubes containing 800 µL of DNA/RNA Shield Stabilization Solution (Zymo Research, Irvine, CA, USA), then kept at −20 °C until processing.

### 2.7. Statistical Analysis

Breed and origin information were obtained from medical records. Descriptive statistics analysis was performed with Microsoft Excel (2016). STATA16.1 (StataCorp, College Station, TX, USA) software was used to fit a logistic regression model assessing the association, expressed as Odds Ratio (OR) between the positivity or negativity to EcPV10 L1 (dependent variable) and four classes of age (group 1: <6 yy; group 2: 6 ≤ 9 yy; group 3: 9 ≤ 13 yy; group 4: ≥13 yy) and two of breed (Thoroughbred vs. the others) (independent variable). Statistical analysis was restricted to mares; the OR was assessed through logistic regression using as dependent variables the positivity/negativity to EcPV10 L1, artificial insemination/natural service, and pluriparous/maiden, while breed and age were considered as independent variables. Finally, a third logistic regression model was fit to assess the OR between being pregnant vs. being empty (dependent variable) and the positivity/negativity to EcPV-10 L1 and fertility/hypo-fertility (independent variables).

## 3. Results

A survey was conducted on the fertility status of a seven-year-old Thoroughbred mare from Chillivani (Sardinia, Italy). Several tests to evaluate the mare fertility were carried out, such as transrectal palpation, ultrasound examination, and vaginal speculum examination, as detailed in Section 2.1, with negative results except for the detection of EcPV2 DNA on Vaginal Cytobrush with a mean Cq of 26.5 ± 0.8. Since our previous data [13] suggested a possible fertility alteration linked to an EcPV2 infection of the genital tract, we decided to sequence this sample to better characterize this case of study.

### 3.1. De Novo Assembly of the Viral Genome

**Figure 1:**
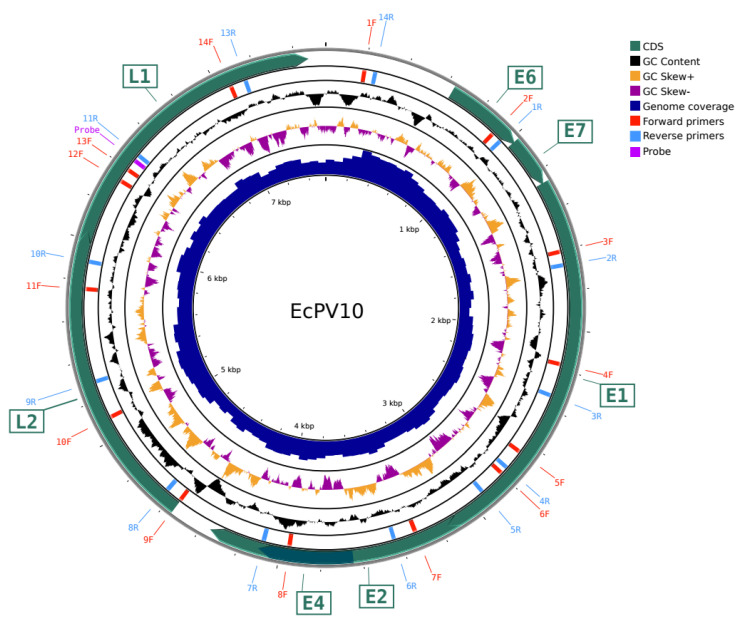
Genomic multi-plot showing the newly assembled EcPV10 genome. The genomic multi-plot was obtained with CGview REF. Concentric circles, from outermost to innermost, show the following: the backbone of the EcPV10 genome; CDS in green arrows; overlapping primers for the genome walking validation method, in forward (red) and reverse orientation (blue); GC content percentage; GC skew, in orange for the forward strand and purple for the reverse strand; Illumina short reads coverage on the assembled sequence.

DNA from three different brush replicates was sequenced and analyzed independently with Next Generation Sequencing (NGS) using Illumina technology. The total amount of raw and filtered reads, as well as the total number of assembled contigs, are summarized in Table S2. The assembled contigs were then aligned using BLASTn towards the in-house built database containing all the EcPV available genomes, listed in Table S1, and four contigs (named in Table S3: NODE_183 of 7543 bp in replicate 1; NODE_467 of 7607 bp in replicate 2; NODE_328 of 5539 bp and NODE_2291 of 1856 bp in replicate 3) were found to be distantly related to EcPV9, EcPV2, EcPV1, and EcPV6, with an average percentage of identity of only 67% (the better alignments of the four contigs are highlighted in yellow in Table S3). Thus, to test the hypothesis that this virus belongs to a new PV genotype, the four contigs were aligned to each other (File S1) to retrieve one single consensus sequence of 7607 bp, further validated with short-reads alignment using BWA [23] and resulting in a 26× average coverage (Figure 1). We analyzed the coding sequence with Gatu [28], PuMA [29], and ORF finder (NCBI) to identify the sequences related to the latent L1 and L2 genes, together with the earlier features E1, E2, E4, E6, and E7 (Figure 1), further supporting our hypothesis that this sequence represents a novel EcPV genotype.

Furthermore, in each replicate, several shorter contigs were related to EcPV2, even with more than 99% of identity highlighted in light green in Table S3, suggesting a double infection, in line with the qPCR results described above, performed using the EcPV2 probe [13].

### 3.2. Sanger Sequencing Validation

The genome sequence for this new PV genotype, named EcPV10, was further validated through Sanger sequencing using a genome walking approach. Primers for overlapping PCR amplicons (Table 1) are schematically represented on the genome sequence in Figure 1. Amplicons were sequenced with Sanger and aligned to the assembled genome sequence obtained from the NGS analysis with the BLASTn algorithm. The final genome obtained through NGS and Sanger sequencing was deposited on the NCBI database under the accession number OP870083, together with the annotation of its features.

### 3.3. Genomic Properties and Phylogenetic Relationships of EcPV10

**Figure 2:**
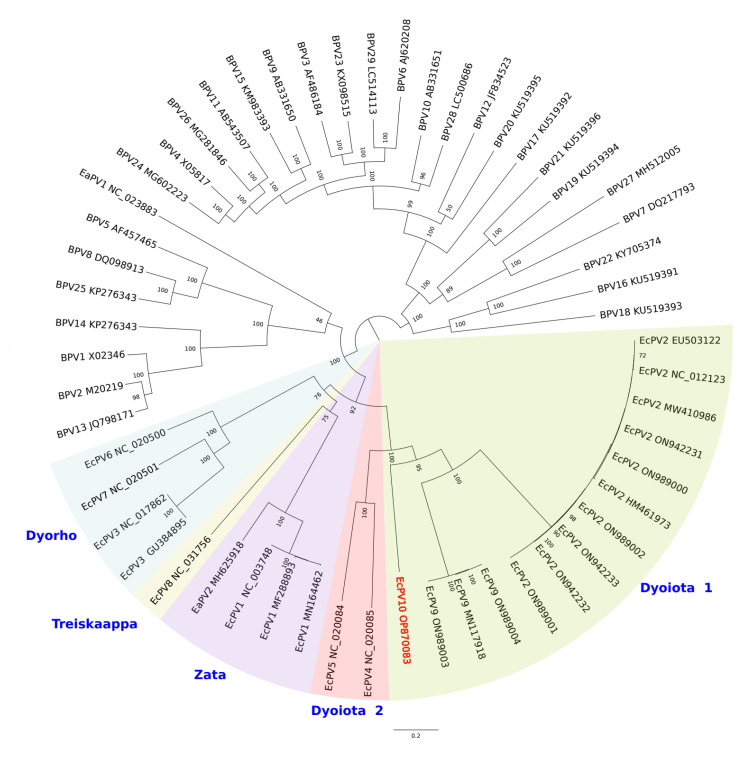
Maximum Likelihood phylogenetic tree based on the concatenated sequences of E1, E2, L2, and L1 genes. Phylogeny was inferred using RaxM. Boostrap values are indicated as node labels. Background colors separate the different EcPV phylogenetic groups from bovine EcPVs, according to [5]. EcPV groups Dyoiota 1 and 2, Dyorho, Treiskaappa, and Zata are indicated in the figure. Our EcPV10 isolate is indicated in red.

The ML phylogenetic analysis based on the concatenated sequences of E1, E2, L2, and L1 showed clustering of EcPV10 among an equine (including horses and donkeys) PV group and a subgroup of bovine PVs (Figure 2). Within the equine PV group, EcPV10 is closely related to EcPV9 and EcPV2 (Dyoiota 1 genus) (Figure 2). These results are in line with the similarity results obtained with BLASTn analysis (Table S3). The Dyoiota 2 genus, including EcPV4 and EcPV5, is the second closest to EcPV10 (Figure 2).

**Figure 3:**
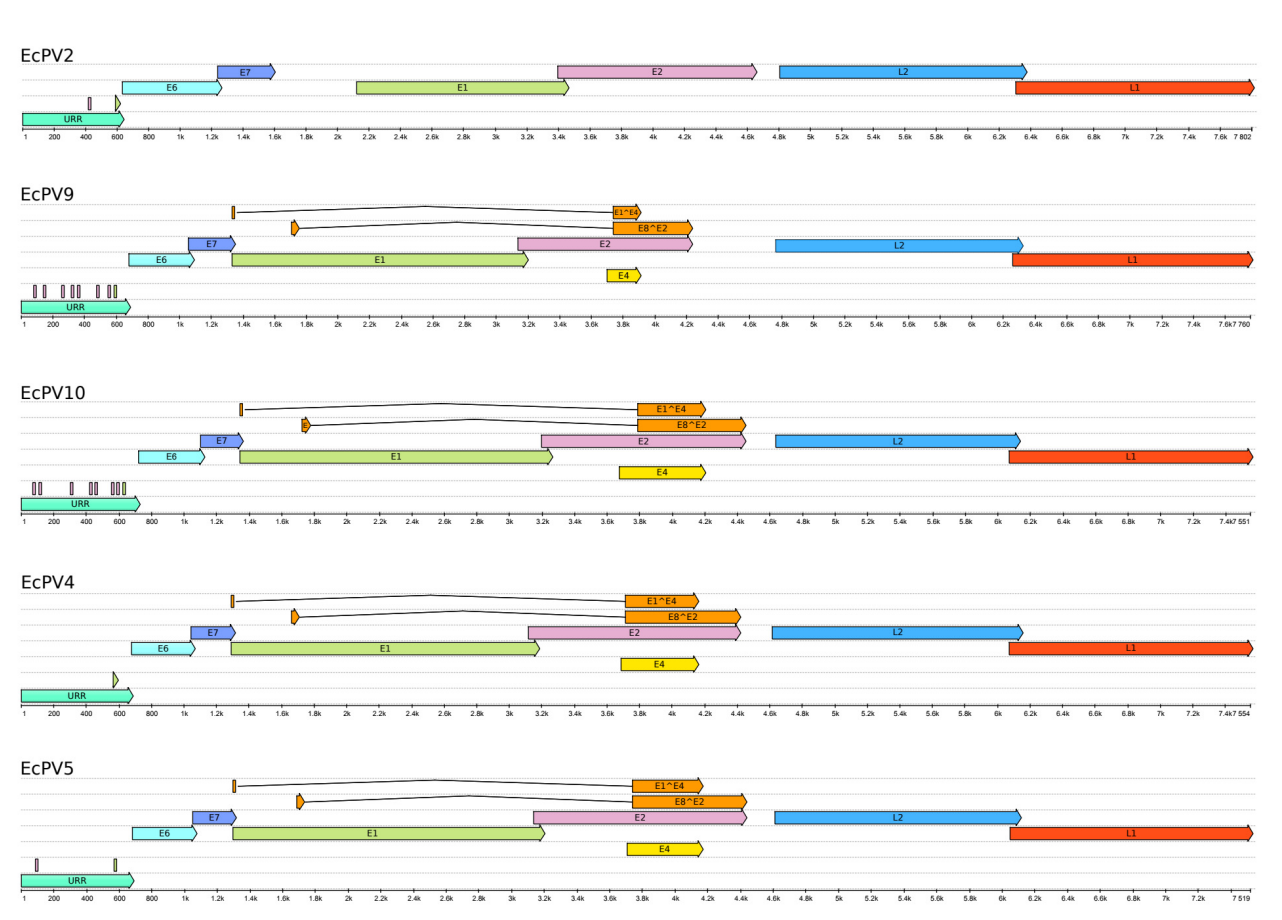
Schematic representation of the closest genomes related to EcPV10. Genomes were annotated with PuMA [29], and each ORFs or regulatory region is indicated with different colors. The picture was obtained with UGENE v36.0 www.ugene.net (accessed on 2 January 2023) and edited with Inkscape v0.92 (www.inkscape.org, accessed on 2 January 2023).

Next, we performed aa pairwise alignments using BLASTp of the six viral ORFs against most closely related PVs (Figure 3). EcPV10 L1, used to classify EcPV [3,16], shared 64.4%, 64.3%, 58.37%, and 60.5% aa similarity with EcPV2, EcPV9, EcPV4, and EcPV5, respectively (Table 2). EcPV10 E1 shared 57.7%, 51.4%, 50.2%, and 48.2% aa similarity with EcPV2, EcPV9, EcPV4, and EcPV5, respectively. The other ORFs shared aa identities between 54.9% and 26.0% with EcPV2, EcPV9, EcPV4, and EcPV5 (Table 2).

### 3.4. EcPV10 Genoprevalence

To investigate the EcPV10 genoprevalence, a total of 216 horses (206 females, 8 stallions, and 2 geldings) were sampled during clinical examination at OVUD (Perugia) and OVU (Turin) and tested for EcPV10 infection using our designed quantitative Real-Time PCR (see Table 1). The age of the sampled horses ranged from 6 months to 22 years, with a mean of 9.8 (±4.6) years and a median of 10 years. Overall, following four categories of age division, 40 animals were <6 yy (very young), 43 were 6 ≤ 9 yy (young), 65 were 9 ≤ 13 yy (adult), and 68 were ≥13 yy (elderly).

The following breeds were analyzed: 91 Standardbred (42.1%), 83 Thoroughbred (38.4%), 12 Italian Sport Horse (5.6%), 8 Arabian (3.7%), 4 Shire (1.9%), 3 Quarter Horse (1.4%), 2 Belgian Sport Horse (0.9%), 1 Appaloosa (0.5%), 1 Maremmano (0.5%), 1 Half Breed (0.5%), and 1 Selle Français (0.5%). The breed has not been reported for 9 animals that were tested.

The samples were obtained from horses from various Italian regions: 144 (67.6%) were from Piedmont, 18 (8.5%) from Umbria, 12 (5.6%) from Tuscany, 9 (4.2%) from Lazio, 4 (2.8%) from Marche, 5 (2.3%) from Emilia Romagna, 5 (2.3%) from Lombardy, 4 (1.8) from Sardinia, 2 (0.9%) from Campania, and 1 (0.5%) from Abruzzo. Additionally, we also examined one sample of horses from France and the USA. The geographical origin was unknown for eight (3.7%) samples.

EcPV10 DNA was found in 3.7% (8 out of 216) of the examined horses, including the first one from Sardinia, with Cq from 30 to 38.7. Seven of the positive subjects were mares, whilst one was a stallion. The age of the positive subjects ranged from 7 to 19 years. Most positive subjects were found in Piedmont (six horses), while the other two were from Sardinia and Tuscany, respectively (Figure S1). Regarding the breed, horses positive for EcPV10 L1 DNA were mostly represented by Thoroughbred (5), followed by Standardbred (1), Quarter horse (1), and one not identified (1) (Table 3).

The logistic regression model did not show any correlation between viral infection risk and age class or breed. Similarly, the presence/absence of the virus did not appear to be related to sex and did not influence fertility in the investigated horses. A larger number of subjects should be examined in future studies in order to confirm our observations.

## 4. Discussion

In this study, we report the discovery, full genome sequence, and classification of EcPV10, a novel *Equus Caballus* Papillomavirus type 10. The complete circular genome of 7607 bp has been deposited on the GenBank database under the accession number OP870083. Total DNA was isolated from a vaginal cytobrush of a mare affected by infertility in triplicate and subjected to different tests, including the screening of EcPV2. Screening for EcPV2 was performed since a recent study [13] suggested a possible negative effect of EcPV2 infection on mares’ fertility, highlighting a pivotal role of EcPV2 infection in the management of horse health. However, the positivity to EcPV2 did not explain the complete clinical picture of the mare, nor her infertility. For this reason, total DNA was sequenced in three different replicates through the application of the Illumina short read technology, using paired-end sequencing methods. The de novo assembly of the raw reads filtered throughout the horse genome confirmed co-infection with EcPV2 and the newly identified EcPV10.

Our phylogenetic analysis demonstrates that EcPV10 is a novel species within the genus DyoiotaPV, most closely related to EcPV2 and EcPV9, and classified as DyoiotaPV1. PVs are classified on the basis of L1, the most conserved gene, but diversity between PV types is enhanced in the E6–E7 oncogene regions [14]. In this respect, L1 protein of EcPV10 shared high aa similarity to EcPV2 and EcPV9, 64.36% and 64.27%, respectively. The rather low similarity was indicative of a novel PV. As for other EcPVs, the ORF encoding for E5 oncogene is not found in EcPV10. This is surprising since E5 is very important for the modulation of host factors and for the control of viral replication and persistence. For example, E5 downregulates MHC I and II expression; however, it shows poor activity in classic transformation assays [33]. Regarding E6 and E7 oncogenes, EcPV10 genes showed high dissimilarity at the nucleotide and aa level when compared with the other EcPVs, as to be expected. Given that these genes are involved in the modulation of cell survival, cellular transcription, cell differentiation, responses to DNA damage, cell cycle progression, and cancer development and progression, it is likely that the genetic differences translate into a different pathogenesis for this virus.

Out of the 216 examined horses using the EcPV10-specific Real Time assay, only 7 mares and 1 stallion tested positive. Despite EcPV10 belonging to Dyoiota 1, the genus that includes the most prevalent PVs within the Italian horse population, i.e., EcPV2 and EcPV9 [3,13], our results indicate a limited circulation of EcPV10 in Italy, to date.

Indeed, EcPV2 and EcPV9 showed a higher infection risk associated with breed compared to EcPV10, with the Thoroughbred being significantly more infected by EcPV2 and EcPV9 compared to other breeds. This finding may be the consequence of natural mating, making sexual transmission the probable way of EcPV2 and EcPV9 diffusion. Risk of spreading EcPV10 was not associated with breed, likely suggesting a different way of transmission for this PV and suggesting no impact on breed upon infection. In this preliminary study, we tested a moderate number of samples (216 horses), which was, however, considered representative enough to suggest a low occurrence of EcPV10 in Italy. Low prevalence of EcPV10 among the Italian horse population could be attributed to several features related to the viral proteins and the capability of the host’s immune response to recognize and eliminate the virus. However, further studies are needed to better understand other possible host–EcPV10 interactions due to the diversity we encountered in its viral proteins. Deeper analyses are also needed in order to shed light into the transmission mechanisms and disease incidence of this new PV isolate.

## Figures and Tables

**Table 1:** Genome walking primers used to verify the reconstructed viral genome.

Primer Number	Sequence 5′-3′		Position (nt)	Expected Product Size (bp)
	Forward	Reverse		
1	AGGTGGAGAGAATGTGCTGC	CGCGTGTATGGTAGAAGGGG	185–981	796
2	GCGGCTACCTGCTTACAGAA	CTACCTGAATGTGGGTCCCG	909–1685	786
3	TCAGCATCAGGACCAGGACA	TGCCAATTTGCTCTCCTCAGT	1596–2346	750
4	CTCCGCCCTGTTCTGGTTTA	TGGTCTCAGCTAGGGGCATA	2163–2768	605
5	GGACCACCAAACACAGGCAA	GGTTGTTTGCCACGTCTACA	2647–2939	292
6	ATGCAACCATACCTGCCTGG	TTAAGCGTGTCCTTAGGCGG	2786–3442	656
7	CCAATGCCAAAACTGCCATCTT	TATCTCGATGCGAAGGAGAAGC	3309–4101	792
8	GCCCAGGGCTACTATTGCATC	TAGGTAGAGGAAGGAGCTGACC	3948–4648	700
9	GTGCATTATGGGGCAGGACT	GGGTCGGAGAGGAAGTTTGG	4545–5301	756
10	TCCGCCTCTGACCATGTGTT	CCGGCATCGATTCAACAACC	5089–5906	817
11	TGTGTCCGTCCTGGACTTTG	AAGCGTGTCTTCCTCCAGTG	5748–6496	748
12 *	GTGTCACAGGTAACCCCCTG	AAGCGTGTCTTCCTCCAGTG	6321–6496	175
13	CAGGTGCCAAGGATGACAGG	GGCATCGGTTGTATGGAGGT	6387–7154	767
14	TGCGACATGTGGAGGAGTTT	GCATTGTTTCGGAAGCCCAG	7056–260	755
Real Time probe	TGCTGGTGGGTTGCAAGCCC		6444–6464	

* Primer pair used in the Real Time assay.

**Table 2:** Percentage of sequence similarity based on amino acid sequences of six ORFs between EcPV10 and EcPV2 (NC_012123), EcPV9 (107-Vigone), EcPV4 (NC_020085), and EcPV5 (NC_020084).

EcPV10	EcPV2	EcPV9	EcPV4	EcPV5
E2	41.20	40.18	36.34	46.91
L2	54.94	46.87	46.43	47.06
L1	64.36	64.27	58.37	60.52
E6	39.84	34.17	47.86	41.23
E7	35.00	50.94	38.46	35.82
E1	57.69	51.38	50.20	48.15

**Table 3:** EcPV10 genoprevalence detected in different breeds.

	EcPV10 Positive	%	EcPV10 Negative	%	Total
Thoroughbred	5	6%	78	94%	83
Standardbred	1	1.1%	90	98.9%	91
Quarter horse	1	33.3%	2	66.7%	3
N.A.	1	11.1%	8	88.9%	9

## Data Availability

The resulting genome of EcPV10 was deposited on the NCBI database under the accession number OP870083.

## Supplementary Materials

The following supporting information can be downloaded at https://www.mdpi.com/article/10.3390/v15030650/s1: Table S1: List of PV and their related accession numbers used for phylogenetic analysis; Table S2: Total amount of raw reads, filtered reads, and number of assembled contigs in the three replicates; Table S3: BLASTn results of the assembled contigs towards the in-house built PV database; File S1: Contigs alignments from the three different replicates; Figure S1: Map of Italy showing the three regions of origin of horses positive for EcPV10. The numbers indicate the number of samples collected in each site.
