# Supplementary material for: Genetic Characterization of a Novel Equus caballus Papillomavirus Isolated from a Thoroughbred Mare
Source: Viruses. 2023 Feb 28;15(3):650. doi: 10.3390/v15030650 (PMC10059215; doi:10.3390/v15030650)
Supplement: Supplementary file 1 [file viruses-15-00650-s001.zip › Figure S1.pdf]

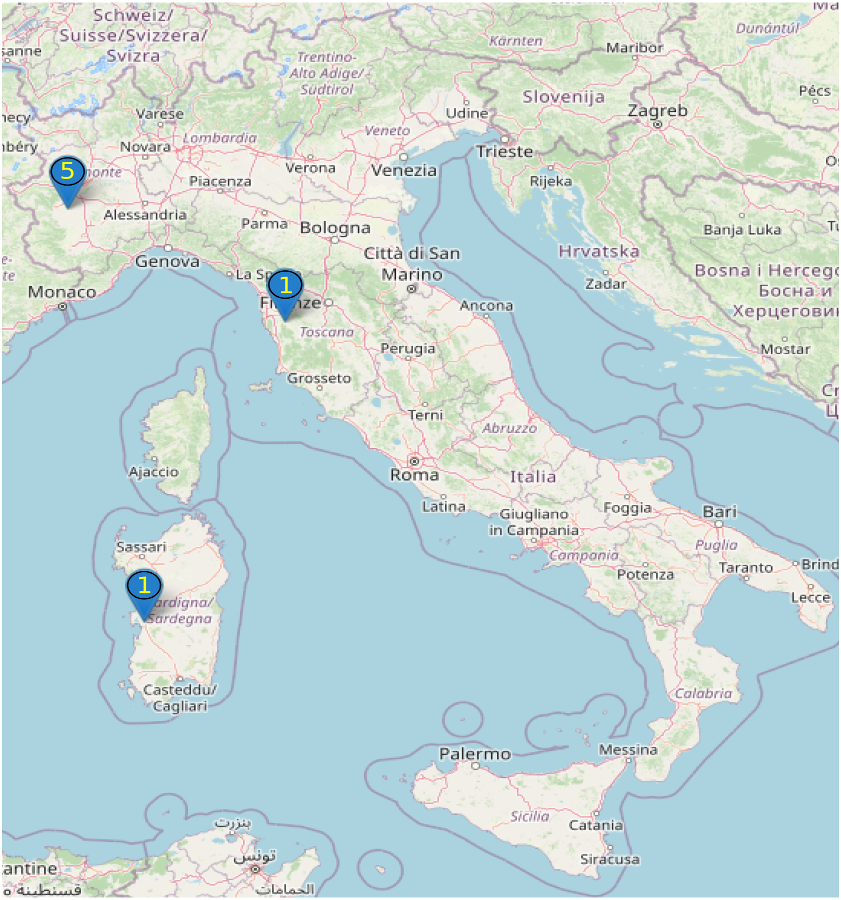

Figure S1: Map of Italy showing the three regions of origin of horses positive for EcPV10. The numbers indicate the number of samples collected in each site.
