# Supplementary material for: Genetic Characterization of a Novel Equus caballus Papillomavirus Isolated from a Thoroughbred Mare
Source: Viruses. 2023 Feb 28;15(3):650. doi: 10.3390/v15030650 (PMC10059215; doi:10.3390/v15030650)
Supplement: Supplementary file 1 [file viruses-15-00650-s001.zip › File S1.pdf]

File S1: Contigs alignments from the three different replicates.

```
RID: NR7Y01K2114
Job Title:NODE_467_length_7607_cov_16.015890
Program: BLASTN
Query: NODE_467_length_7607_cov_16.015890 ID: lcl|Query_45867(dna) Length: 7607
Subject:NODE_328_length_5539_cov_3.362871 ID: lcl|Query_45869(dna) Length: 5539

Sequences producing significant alignments:
                                                                  Scientific
Common                     Max    Total Query   E   Per.   Acc.
Description                                                       Name
Name            Taxid      Score  Score cover Value Ident  Len        Accession
NODE_328_length_5539_cov_3.362871
0          5925   10315 73%   0.0   99.91  5539       Query_45869

Alignments:

>NODE_328_length_5539_cov_3.362871
Sequence ID: Query_45869 Length: 5539
Range 1: 2323 to 5539

Score:5925 bits(3208), Expect:0.0,
Identities:3214/3217(99%),  Gaps:0/3217(0%), Strand: Plus/Plus

Query  1     cccccccccAGGGAAGCGGCGGCGCCGCGCCGCGGAGGGTGACCTGTACCGAGGGTGCAT  60
             ||||||||||||||||||||||||||||||||||||||||||||||||||||||||||||
Sbjct  2323  CCCCCCCCCAGGGAAGCGGCGGCGCCGCGCCGCGGAGGGTGACCTGTACCGAGGGTGCAT  2382

Query  61    TATGGGGCAGGACTGCCCGCCGGACATCAAGGCAAAGTTTGAGAACGACACACTTGCAGA  120
             ||||||||||||||||||||||||||||||||||||||||||||||||||||||||||||
Sbjct  2383  TATGGGGCAGGACTGCCCGCCGGACATCAAGGCAAAGTTTGAGAACGACACACTTGCAGA  2442

Query  121   CCGCTTTCTAAAGTGGGTCAGCTCCTTCCTCTACCTAGGCAACCTGGGTTTCAGCACGGG  180
             ||||||||||||||||||||||||||||||||||||||||||||||||||||||||||||
Sbjct  2443  CCGCTTTCTAAAGTGGGTCAGCTCCTTCCTCTACCTAGGCAACCTGGGTTTCAGCACGGG  2502

Query  181   CAGGGGCAATCCCAGGTTTGGGTACAGACCTGTggggggcggggtaggcgaggcgggtgg  240
             ||||||||||||||||||||||||||||||||||||||||||||||||||||||||| ||
Sbjct  2503  CAGGGGCAATCCCAGGTTTGGGTACAGACCTGTGGGGGGCGGGGTAGGCGAGGCGGGGGG  2562

Query  241   gggggtgcgggtcgggACGCCCATTACGGACGTGTCCCCGGGGGTCACTGTGGAGACGGT  300
             ||||| ||||||||||||||||||||||||||||||||||||||||||||||||||||||
Sbjct  2563  GGGGGGGCGGGTCGGGACGCCCATTACGGACGTGTCCCCGGGGGTCACTGTGGAGACGGT  2622

Query  301   GGGGCCCGCGGAGGTGGTCCCAGTAGACAGTTTAAGCCCCTCAGCCCCGGCTGTGGTGCC  360
             ||||||||||||||||||||||||||||||||||||||||||||||||||||||||||||
Sbjct  2623  GGGGCCCGCGGAGGTGGTCCCAGTAGACAGTTTAAGCCCCTCAGCCCCGGCTGTGGTGCC  2682

Query  361   TGCGGGGGAAGGCACAGGGGGCGCTGTGGATGTGGAGTTGGTGGCTGAGGTGCACCCTAC  420
             ||||||||||||||||||||||||||||||||||||||||||||||||||||||||||||
Sbjct  2683  TGCGGGGGAAGGCACAGGGGGCGCTGTGGATGTGGAGTTGGTGGCTGAGGTGCACCCTAC  2742

Query  421   CGTGGACCCGGGCATGGCTGGGGGCTCGGGGGGTGTTACCAGCAACGGCGGGGATGCCGC  480
             ||||||||||||||||||||||||||||||||||||||||||||||||||||||||||||
Sbjct  2743  CGTGGACCCGGGCATGGCTGGGGGCTCGGGGGGTGTTACCAGCAACGGCGGGGATGCCGC  2802

Query  481   CGTGCTGGAGGTGCCCCCTGAGGTGGTTCCCCGGGGTCGGAATGCTACTAGCCGCACACA  540
             ||||||||||||||||||||||||||||||||||||||||||||||||||||||||||||
Sbjct  2803  CGTGCTGGAGGTGCCCCCTGAGGTGGTTCCCCGGGGTCGGAATGCTACTAGCCGCACACA  2862
```

```
Query  541   GTTTCATAATCCTGCATTCCATGTGGAGCTAAACAGCTCGCTCCCCACGGGTGAATCGTC  600
             ||||||||||||||||||||||||||||||||||||||||||||||||||||||||||||
Sbjct  2863  GTTTCATAATCCTGCATTCCATGTGGAGCTAAACAGCTCGCTCCCCACGGGTGAATCGTC  2922

Query  601   CGCCTCTGACCATGTGTTTGTCCAGGCGGAACAGGGTGGTCACTGGGTGGGAGAGGAAAT  660
             ||||||||||||||||||||||||||||||||||||||||||||||||||||||||||||
Sbjct  2923  CGCCTCTGACCATGTGTTTGTCCAGGCGGAACAGGGTGGTCACTGGGTGGGAGAGGAAAT  2982

Query  661   TGAATTGCTTCCTTTGGGCGATACGCTGACGCAAAGAACCAGCACCCCCAGAACTGGGGC  720
             ||||||||||||||||||||||||||||||||||||||||||||||||||||||||||||
Sbjct  2983  TGAATTGCTTCCTTTGGGCGATACGCTGACGCAAAGAACCAGCACCCCCAGAACTGGGGC  3042

Query  721   ACGGGGACGCGCAGAGAGCGGGGCGGAGCGTCTATTTGGACGTGGCTTCCGTCAGGTGCG  780
             ||||||||||||||||||||||||||||||||||||||||||||||||||||||||||||
Sbjct  3043  ACGGGGACGCGCAGAGAGCGGGGCGGAGCGTCTATTTGGACGTGGCTTCCGTCAGGTGCG  3102

Query  781   CGTAACCGATCCAAACTTCCTCTCCGACCCTGGGTCTCTTGTGCAGTTTGGGTTCGAGAA  840
             ||||||||||||||||||||||||||||||||||||||||||||||||||||||||||||
Sbjct  3103  CGTAACCGATCCAAACTTCCTCTCCGACCCTGGGTCTCTTGTGCAGTTTGGGTTCGAGAA  3162

Query  841   CCCTGCATATGACCCCTCCGGCAGCATCTCCTTTGGGCCACTCGGCGAGCCGGAGGCGGC  900
             ||||||||||||||||||||||||||||||||||||||||||||||||||||||||||||
Sbjct  3163  CCCTGCATATGACCCCTCCGGCAGCATCTCCTTTGGGCCACTCGGCGAGCCGGAGGCGGC  3222

Query  901   CCCCAACCCAGAGTTTCAGGATGTTGTGCACCTTGGCAGGACACACATTACAGAGCGCGA  960
             ||||||||||||||||||||||||||||||||||||||||||||||||||||||||||||
Sbjct  3223  CCCCAACCCAGAGTTTCAGGATGTTGTGCACCTTGGCAGGACACACATTACAGAGCGCGA  3282

Query  961   CGGGCGCGTGGTGCTAGGAAGATTTGGTCAGCGGGCTGGGGTGTCCACCCGCAGCGGGCG  1020
             ||||||||||||||||||||||||||||||||||||||||||||||||||||||||||||
Sbjct  3283  CGGGCGCGTGGTGCTAGGAAGATTTGGTCAGCGGGCTGGGGTGTCCACCCGCAGCGGGCG  3342

Query  1021  GGTGGTGGGACCCCAGGTACACTATAGGTTTGAGTTCAGCAGCATTGCCCCTGCAGAGGA  1080
             ||||||||||||||||||||||||||||||||||||||||||||||||||||||||||||
Sbjct  3343  GGTGGTGGGACCCCAGGTACACTATAGGTTTGAGTTCAGCAGCATTGCCCCTGCAGAGGA  3402

Query  1081  GATTGAACTGGTGCCTCTGCCAAGTGCGGATCCGCCGGGGTCACTTGGGGACGAGTTTGA  1140
             ||||||||||||||||||||||||||||||||||||||||||||||||||||||||||||
Sbjct  3403  GATTGAACTGGTGCCTCTGCCAAGTGCGGATCCGCCGGGGTCACTTGGGGACGAGTTTGA  3462

Query  1141  GGTTATCGACCTGGACAGCACAGGTAGTGTGTACAGCGAGGCGGCTCTGCTGGACGATGA  1200
             ||||||||||||||||||||||||||||||||||||||||||||||||||||||||||||
Sbjct  3463  GGTTATCGACCTGGACAGCACAGGTAGTGTGTACAGCGAGGCGGCTCTGCTGGACGATGA  3522

Query  1201  CAGCCTCAGCATTCACGGGGTGCTTAGCTTGGGTCAGAGAGCTGGCAGCCACCGCGCTGT  1260
             ||||||||||||||||||||||||||||||||||||||||||||||||||||||||||||
Sbjct  3523  CAGCCTCAGCATTCACGGGGTGCTTAGCTTGGGTCAGAGAGCTGGCAGCCACCGCGCTGT  3582

Query  1261  GTCCGTCCTGGACTTTGCTGGACCTAGAGCCTTTTACAGTGCACTGACAGTAGAGGGCGA  1320
             ||||||||||||||||||||||||||||||||||||||||||||||||||||||||||||
Sbjct  3583  GTCCGTCCTGGACTTTGCTGGACCTAGAGCCTTTTACAGTGCACTGACAGTAGAGGGCGA  3642

Query  1321  CGTGGCACCTGCACACCCGAACCCCACTAGCTCTATCCTTGTGGTCCCTGAGAACGCCGA  1380
             ||||||||||||||||||||||||||||||||||||||||||||||||||||||||||||
Sbjct  3643  CGTGGCACCTGCACACCCGAACCCCACTAGCTCTATCCTTGTGGTCCCTGAGAACGCCGA  3702

Query  1381  GCAGACCCCCTCTGTGGTTGTTGAATCGATGCCGGTCAGTGGCCTGTATGACCTGCACCC  1440
             ||||||||||||||||||||||||||||||||||||||||||||||||||||||||||||
Sbjct  3703  GCAGACCCCCTCTGTGGTTGTTGAATCGATGCCGGTCAGTGGCCTGTATGACCTGCACCC  3762

Query  1441  CAGCTTGCACCCTAGACGCAGGCGCAAACGCAGGCGTTCCTTCTGTTTTGCAGATGGCAG  1500
             ||||||||||||||||||||||||||||||||||||||||||||||||||||||||||||
Sbjct  3763  CAGCTTGCACCCTAGACGCAGGCGCAAACGCAGGCGTTCCTTCTGTTTTGCAGATGGCAG  3822
```

```
Query  1501  TGTGGACACAAAACAAGCAGAAGCTTTTTCTGCCTCCGCCGCCTGTTACTAGAGTCCCTA  1560
             ||||||||||||||||||||||||||||||||||||||||||||||||||||||||||||
Sbjct  3823  TGTGGACACAAAACAAGCAGAAGCTTTTTCTGCCTCCGCCGCCTGTTACTAGAGTCCCTA  3882

Query  1561  ACACAGATGAGTATGTCGTGCGACACAACATATTTTACCATGGTAACAGTGACCGACTGC  1620
             ||||||||||||||||||||||||||||||||||||||||||||||||||||||||||||
Sbjct  3883  ACACAGATGAGTATGTCGTGCGACACAACATATTTTACCATGGTAACAGTGACCGACTGC  3942

Query  1621  TTATGGTCGGCCACCCTTTCTTTCCTGTGAAGAGTGGCAACATAGATGTTCCTAAGGTGT  1680
             ||||||||||||||||||||||||||||||||||||||||||||||||||||||||||||
Sbjct  3943  TTATGGTCGGCCACCCTTTCTTTCCTGTGAAGAGTGGCAACATAGATGTTCCTAAGGTGT  4002

Query  1681  CTGCAAATCAATTCAGGGTCTTTAAGGTGCTTCTGCCTGACCCTAATAAGTTTGCTCTGG  1740
             ||||||||||||||||||||||||||||||||||||||||||||||||||||||||||||
Sbjct  4003  CTGCAAATCAATTCAGGGTCTTTAAGGTGCTTCTGCCTGACCCTAATAAGTTTGCTCTGG  4062

Query  1741  CCGACCCTGACTTATACAACCCGGAGACGGGCCGCCTGGTATGGGCCCTAAAGGGCTTGG  1800
             ||||||||||||||||||||||||||||||||||||||||||||||||||| ||||||||
Sbjct  4063  CCGACCCTGACTTATACAACCCGGAGACGGGCCGCCTGGTATGGGCCCTAACGGGCTTGG  4122

Query  1801  AGGTTAGCAGGGGCCAGCCCCTGGGCATAGGTGTCACAGGTAACCCCCTGTTTAACAAAA  1860
             ||||||||||||||||||||||||||||||||||||||||||||||||||||||||||||
Sbjct  4123  AGGTTAGCAGGGGCCAGCCCCTGGGCATAGGTGTCACAGGTAACCCCCTGTTTAACAAAA  4182

Query  1861  AGAATGATGTTGAAAACCCTAGCAAGCTACAGGGGGCAGGTGCCAAGGATGACAGGGTGA  1920
             ||||||||||||||||||||||||||||||||||||||||||||||||||||||||||||
Sbjct  4183  AGAATGATGTTGAAAACCCTAGCAAGCTACAGGGGGCAGGTGCCAAGGATGACAGGGTGA  4242

Query  1921  ACATGGGCTTTGATGTGAAGCAACACCAGCTGCTGCTGGTGGGTTGCAAGCCCCCACAGG  1980
             ||||||||||||||||||||||||||||||||||||||||||||||||||||||||||||
Sbjct  4243  ACATGGGCTTTGATGTGAAGCAACACCAGCTGCTGCTGGTGGGTTGCAAGCCCCCACAGG  4302

Query  1981  GGGAGCACTGGAGGAAGACACGCTTTTGCAACACAGGACAGCCACCGGTGGGCTCCTGCC  2040
             ||||||||||||||||||||||||||||||||||||||||||||||||||||||||||||
Sbjct  4303  GGGAGCACTGGAGGAAGACACGCTTTTGCAACACAGGACAGCCACCGGTGGGCTCCTGCC  4362

Query  2041  CGGCCATAGAGCTGGTCAATACCGTGATAGAGGATGGGGACATGGGGGACGTGGGTTTCG  2100
             ||||||||||||||||||||||||||||||||||||||||||||||||||||||||||||
Sbjct  4363  CGGCCATAGAGCTGGTCAATACCGTGATAGAGGATGGGGACATGGGGGACGTGGGTTTCG  4422

Query  2101  GGGCAATGGACTTTGCCACACTTTGTGACAGTAAGGCCGATGTGCCTCTGGACCTCGTGG  2160
             ||||||||||||||||||||||||||||||||||||||||||||||||||||||||||||
Sbjct  4423  GGGCAATGGACTTTGCCACACTTTGTGACAGTAAGGCCGATGTGCCTCTGGACCTCGTGG  4482

Query  2161  GCACTGCCTCGAAGTACCCCGACTACATTAAGATGGGGCAGGAGCCTGCAGGGGACAGCA  2220
             ||||||||||||||||||||||||||||||||||||||||||||||||||||||||||||
Sbjct  4483  GCACTGCCTCGAAGTACCCCGACTACATTAAGATGGGGCAGGAGCCTGCAGGGGACAGCA  4542

Query  2221  TGTGGTTTTTCGCCAGGAGGGAGCAGTACTATGCCAGGCACTTTTTTACGCGGGACGGTA  2280
             ||||||||||||||||||||||||||||||||||||||||||||||||||||||||||||
Sbjct  4543  TGTGGTTTTTCGCCAGGAGGGAGCAGTACTATGCCAGGCACTTTTTTACGCGGGACGGTA  4602

Query  2281  AGAGTCTGGAGACGGTGCCCCCCGAGTTGTACACAGCACCAGAGGCAAACCCAACCAACA  2340
             ||||||||||||||||||||||||||||||||||||||||||||||||||||||||||||
Sbjct  4603  AGAGTCTGGAGACGGTGCCCCCCGAGTTGTACACAGCACCAGAGGCAAACCCAACCAACA  4662

Query  2341  TCAACAGGTACATTTACAGTGCAAGCCCCAGTGGGTCTCTCGTGTCCACAGACTCACAGA  2400
             ||||||||||||||||||||||||||||||||||||||||||||||||||||||||||||
Sbjct  4663  TCAACAGGTACATTTACAGTGCAAGCCCCAGTGGGTCTCTCGTGTCCACAGACTCACAGA  4722

Query  2401  TATTTAACAGGCCGTACTGGCTGGCACGCGCACAGGGCCAGAATAATGGCATCTGCTGGC  2460
             ||||||||||||||||||||||||||||||||||||||||||||||||||||||||||||
Sbjct  4723  TATTTAACAGGCCGTACTGGCTGGCACGCGCACAGGGCCAGAATAATGGCATCTGCTGGC  4782
```

```
Query  2461  ACAACACTTTGTATGTGACCGTGTTTGACAACACACGTGGCACAAACTTGACCATTACAG  2520
             ||||||||||||||||||||||||||||||||||||||||||||||||||||||||||||
Sbjct  4783  ACAACACTTTGTATGTGACCGTGTTTGACAACACACGTGGCACAAACTTGACCATTACAG  4842

Query  2521  TGAACCCCCAGAACACATATGATTCCACAGGCTTTAATGCATACGTGCGACATGTGGAGG  2580
             ||||||||||||||||||||||||||||||||||||||||||||||||||||||||||||
Sbjct  4843  TGAACCCCCAGAACACATATGATTCCACAGGCTTTAATGCATACGTGCGACATGTGGAGG  4902

Query  2581  AGTTTGAGCTCTCCTTTATCTTTCAGCTGTGCACGGTCCCCTTGACACCCCAGGTCCTTG  2640
             ||||||||||||||||||||||||||||||||||||||||||||||||||||||||||||
Sbjct  4903  AGTTTGAGCTCTCCTTTATCTTTCAGCTGTGCACGGTCCCCTTGACACCCCAGGTCCTTG  4962

Query  2641  CCCACCTCCATACAACCGATGCCAGCCTCCTTGAGGAGTGGGAGATTGGGGTAAAccccc  2700
             ||||||||||||||||||||||||||||||||||||||||||||||||||||||||||||
Sbjct  4963  CCCACCTCCATACAACCGATGCCAGCCTCCTTGAGGAGTGGGAGATTGGGGTAAACCCCC  5022

Query  2701  ccTCCTCCTCGCAGCTGGAGGACACATACCGGTTTATTACCTCTACTGCCACAAAGTGCC  2760
             ||||||||||||||||||||||||||||||||||||||||||||||||||||||||||||
Sbjct  5023  CCTCCTCCTCGCAGCTGGAGGACACATACCGGTTTATTACCTCTACTGCCACAAAGTGCC  5082

Query  2761  CTGTGCCTCCCCAGGCCCCGCTTGAGCCACCGGGGTACACATTTTGGACTGTGGATCTGC  2820
             ||||||||||||||||||||||||||||||||||||||||||||||||||||||||||||
Sbjct  5083  CTGTGCCTCCCCAGGCCCCGCTTGAGCCACCGGGGTACACATTTTGGACTGTGGATCTGC  5142

Query  2821  AGGAGCGTTTGTCCTTGGACCTGGACCAGTACACCCTGGGGAGGCGCTTTTTGGCGCAGT  2880
             ||||||||||||||||||||||||||||||||||||||||||||||||||||||||||||
Sbjct  5143  AGGAGCGTTTGTCCTTGGACCTGGACCAGTACACCCTGGGGAGGCGCTTTTTGGCGCAGT  5202

Query  2881  CAGGGGTGTCTACCACACGCTCGCTTAGGGCCCCCACCTCCAGAAAGCGCAAGTCATCAG  2940
             ||||||||||||||||||||||||||||||||||||||||||||||||||||||||||||
Sbjct  5203  CAGGGGTGTCTACCACACGCTCGCTTAGGGCCCCCACCTCCAGAAAGCGCAAGTCATCAG  5262

Query  2941  GGTCGGCCACCAGAAGGACCACCAAAAGAAGGAAATAGTGTGTATGCCATTCTGTTTTTC  3000
             ||||||||||||||||||||||||||||||||||||||||||||||||||||||||||||
Sbjct  5263  GGTCGGCCACCAGAAGGACCACCAAAAGAAGGAAATAGTGTGTATGCCATTCTGTTTTTC  5322

Query  3001  ATATTTAATAAAGACTTTTGAATAAGCACTGTGTCATTCATGTTCATGCACCGCACCCGG  3060
             ||||||||||||||||||||||||||||||||||||||||||||||||||||||||||||
Sbjct  5323  ATATTTAATAAAGACTTTTGAATAAGCACTGTGTCATTCATGTTCATGCACCGCACCCGG  5382

Query  3061  TGCAAGTATGCACCGCCTGGGATATATACCGTCCGCGGTGCCATCGCACCTGGTGCTCAC  3120
             ||||||||||||||||||||||||||||||||||||||||||||||||||||||||||||
Sbjct  5383  TGCAAGTATGCACCGCCTGGGATATATACCGTCCGCGGTGCCATCGCACCTGGTGCTCAC  5442

Query  3121  TTCGTGCCAGCTGTACCATTGCCATGGAGGCAGAGGATTAAGGTAAGTGCTTTTAAATCC  3180
             ||||||||||||||||||||||||||||||||||||||||||||||||||||||||||||
Sbjct  5443  TTCGTGCCAGCTGTACCATTGCCATGGAGGCAGAGGATTAAGGTAAGTGCTTTTAAATCC  5502

Query  3181  TATGtttttttttCTCCATTGTTTAACTGCACAGTTT  3217
             |||||||||||||||||||||||||||||||||||||
Sbjct  5503  TATGTTTTTTTTTCTCCATTGTTTAACTGCACAGTTT  5539
```

Range 2: 1 to 2377

Score:4390 bits(2377), Expect:0.0,
Identities:2377/2377(100%), Gaps:0/2377(0%), Strand: Plus/Plus

```
Query  5231  CTGTTCTGGTTTAAGGGCAGCATGTCCAATTGTACATTTACACATGGGATTATGCCGCAG  5290
             ||||||||||||||||||||||||||||||||||||||||||||||||||||||||||||
Sbjct  1     CTGTTCTGGTTTAAGGGCAGCATGTCCAATTGTACATTTACACATGGGATTATGCCGCAG  60
```

```
Query  5291  TGGCTGCGAGCACAAACCATGCTTAGCCAACAGACTGAGGAGGCGGTCAAATTTGACCTG  5350
             ||||||||||||||||||||||||||||||||||||||||||||||||||||||||||||
Sbjct  61    TGGCTGCGAGCACAAACCATGCTTAGCCAACAGACTGAGGAGGCGGTCAAATTTGACCTG  120

Query  5351  TCAGACATGATACAGTGGGCGCTGGACAATGACATAACTGAGGAGAGCAAATTGGCATAT  5410
             ||||||||||||||||||||||||||||||||||||||||||||||||||||||||||||
Sbjct  121   TCAGACATGATACAGTGGGCGCTGGACAATGACATAACTGAGGAGAGCAAATTGGCATAT  180

Query  5411  GGGTACGCCCTCCTGGCCGAGAGCGACCCCAATGCAGCCGCCTTCCTGTCCTCCAACAAC  5470
             ||||||||||||||||||||||||||||||||||||||||||||||||||||||||||||
Sbjct  181   GGGTACGCCCTCCTGGCCGAGAGCGACCCCAATGCAGCCGCCTTCCTGTCCTCCAACAAC  240

Query  5471  CAGGCAAAACACGTGCGTGACGCGGCCACTATGGTCCGCCATTACAAGCGGGCACAGATG  5530
             ||||||||||||||||||||||||||||||||||||||||||||||||||||||||||||
Sbjct  241   CAGGCAAAACACGTGCGTGACGCGGCCACTATGGTCCGCCATTACAAGCGGGCACAGATG  300

Query  5531  CTGACCATGTCAATGTCCGCATGGGTGCACAGGCGGTGTGAGGCGGTGGAGGAGCAAGGG  5590
             ||||||||||||||||||||||||||||||||||||||||||||||||||||||||||||
Sbjct  301   CTGACCATGTCAATGTCCGCATGGGTGCACAGGCGGTGTGAGGCGGTGGAGGAGCAAGGG  360

Query  5591  GACTGGCGACCAATTATTTATTTCTGAAATATCAGAATGTGGAAATTGCTGCATTTTTG  5650
             ||||||||||||||||||||||||||||||||||||||||||||||||||||||||||||
Sbjct  361   GACTGGCGACCAATTATTTATTTCTGAAATATCAGAATGTGGAAATTGCTGCATTTTTG  420

Query  5651  CAGTGCCTCAAACAGTTCTTTAAAGGGGTCCCCAAAAGAAATTGTCTAGTAATCCAGGGA  5710
             ||||||||||||||||||||||||||||||||||||||||||||||||||||||||||||
Sbjct  421   CAGTGCCTCAAACAGTTCTTTAAAGGGGTCCCCAAAAGAAATTGTCTAGTAATCCAGGGA  480

Query  5711  CCACCAAACACAGGCAAGTCCACCTTCTGTATGAGCCTCATCAATTTCCTTGCAGGGAAG  5770
             ||||||||||||||||||||||||||||||||||||||||||||||||||||||||||||
Sbjct  481   CCACCAAACACAGGCAAGTCCACCTTCTGTATGAGCCTCATCAATTTCCTTGCAGGGAAG  540

Query  5771  GTGCTGTCCTTTGTTAATAGCAGGAGCCAGTTCTGGCTTATGCCCCTAGCTGAGACCAAG  5830
             ||||||||||||||||||||||||||||||||||||||||||||||||||||||||||||
Sbjct  541   GTGCTGTCCTTTGTTAATAGCAGGAGCCAGTTCTGGCTTATGCCCCTAGCTGAGACCAAG  600

Query  5831  GTGGCGCTGTTGGACGATGCAACCATACCTGCCTGGGATTACCTAGACACATTCATGAGG  5890
             ||||||||||||||||||||||||||||||||||||||||||||||||||||||||||||
Sbjct  601   GTGGCGCTGTTGGACGATGCAACCATACCTGCCTGGGATTACCTAGACACATTCATGAGG  660

Query  5891  AATGCCATGGACGGGAACCCCATCTGTATAGACCTGAAGCATCGGGCGCCCGTCCAAACA  5950
             ||||||||||||||||||||||||||||||||||||||||||||||||||||||||||||
Sbjct  661   AATGCCATGGACGGGAACCCCATCTGTATAGACCTGAAGCATCGGGCGCCCGTCCAAACA  720

Query  5951  CGCTGCCCCCCTTTGTTAGTAACCACTAATGTAGACGTGGCAAACAACCCCAGATATCAA  6010
             ||||||||||||||||||||||||||||||||||||||||||||||||||||||||||||
Sbjct  721   CGCTGCCCCCCTTTGTTAGTAACCACTAATGTAGACGTGGCAAACAACCCCAGATATCAA  780

Query  6011  TACCTCCACACACGGATGGCCTGTTTCACCTTTCCTAATGACTTTATCTTAGATGAGAAT  6070
             ||||||||||||||||||||||||||||||||||||||||||||||||||||||||||||
Sbjct  781   TACCTCCACACACGGATGGCCTGTTTCACCTTTCCTAATGACTTTATCTTAGATGAGAAT  840

Query  6071  GGGCAGCCTGTGCACCAACTAACTGTAGCCAATTGGGCCTGTTTTTTCAAAAGGTTGTGG  6130
             ||||||||||||||||||||||||||||||||||||||||||||||||||||||||||||
Sbjct  841   GGGCAGCCTGTGCACCAACTAACTGTAGCCAATTGGGCCTGTTTTTTCAAAAGGTTGTGG  900

Query  6131  AGCAGATTAGAGTTCAGTGAACAGGAAGACGAGGGCGAAGATGGAGAAGCTCCACAGACA  6190
             ||||||||||||||||||||||||||||||||||||||||||||||||||||||||||||
Sbjct  901   AGCAGATTAGAGTTCAGTGAACAGGAAGACGAGGGCGAAGATGGAGAAGCTCCACAGACA  960

Query  6191  CTTAGAGTCTGTTACAGAAAGGCAGATGGAGATGTATGAAAGGGGCAGCACACGCCTGAT  6250
             ||||||||||||||||||||||||||||||||||||||||||||||||||||||||||||
Sbjct  961   CTTAGAGTCTGTTACAGAAAGGCAGATGGAGATGTATGAAAGGGGCAGCACACGCCTGAT  1020
```

```
Query  6251  TGATCAGGTGGAGCACTGGACGCTGCAAAGGAGAGAGGCTGTGCTTCTGCATGCTGCCAG  6310
             ||||||||||||||||||||||||||||||||||||||||||||||||||||||||||||
Sbjct  1021  TGATCAGGTGGAGCACTGGACGCTGCAAAGGAGAGAGGCTGTGCTTCTGCATGCTGCCAG  1080

Query  6311  GAAGCGGGGCCTACACAGAGTTGGGCCATTTCCGGTGCCCAATGCGGCCACCTCAGCTGC  6370
             ||||||||||||||||||||||||||||||||||||||||||||||||||||||||||||
Sbjct  1081  GAAGCGGGGCCTACACAGAGTTGGGCCATTTCCGGTGCCCAATGCGGCCACCTCAGCTGC  1140

Query  6371  CAATGCCAAAACTGCCATCTTCATGCAGCTGACCCTTGAGTCGCTCCTCCACTCGCAGTT  6430
             ||||||||||||||||||||||||||||||||||||||||||||||||||||||||||||
Sbjct  1141  CAATGCCAAAACTGCCATCTTCATGCAGCTGACCCTTGAGTCGCTCCTCCACTCGCAGTT  1200

Query  6431  TGCAGAGGAGCCCTGGACCATGTCAGAGACCACAGCTGACATGTTTAACTGCCCGCCTAA  6490
             ||||||||||||||||||||||||||||||||||||||||||||||||||||||||||||
Sbjct  1201  TGCAGAGGAGCCCTGGACCATGTCAGAGACCACAGCTGACATGTTTAACTGCCCGCCTAA  1260

Query  6491  GGACACGCTTAAAAAGGACGGCTTTGTTGTGCAGGTGAAATTTGACAAGCAGGACGAAAA  6550
             ||||||||||||||||||||||||||||||||||||||||||||||||||||||||||||
Sbjct  1261  GGACACGCTTAAAAAGGACGGCTTTGTTGTGCAGGTGAAATTTGACAAGCAGGACGAAAA  1320

Query  6551  CAGGATGTGGTACACAGCATGGGGCAGCATTTACTACAGGAACCTTGAGGGGGACTGGGT  6610
             ||||||||||||||||||||||||||||||||||||||||||||||||||||||||||||
Sbjct  1321  CAGGATGTGGTACACAGCATGGGGCAGCATTTACTACAGGAACCTTGAGGGGGACTGGGT  1380

Query  6611  GAAGACGCAGGGTAAGGCGAACGCCCTGGGCCTCTACTATGAGGCGGACGGGGaaaaaaa  6670
             ||||||||||||||||||||||||||||||||||||||||||||||||||||||||||||
Sbjct  1381  GAAGACGCAGGGTAAGGCGAACGCCCTGGGCCTCTACTATGAGGCGGACGGGGAAAAAAA  1440

Query  6671  aTACTATGTACAGTTTGGTGATGAGGCCGCAAAGTATGGCACCTCCAGAAGCTGGGAGGT  6730
             ||||||||||||||||||||||||||||||||||||||||||||||||||||||||||||
Sbjct  1441  ATACTATGTACAGTTTGGTGATGAGGCCGCAAAGTATGGCACCTCCAGAAGCTGGGAGGT  1500

Query  6731  TTCCTATCGCACTGAAACCTTCACCTATCCTTTCAACAGCGGATGCGGTGCAGCCGGACC  6790
             ||||||||||||||||||||||||||||||||||||||||||||||||||||||||||||
Sbjct  1501  TTCCTATCGCACTGAAACCTTCACCTATCCTTTCAACAGCGGATGCGGTGCAGCCGGACC  1560

Query  6791  CACAGAACCGATTCCAGGAGGACAGCCCACACCTCCAGCTACCCCTGACTCGCCGGGACT  6850
             ||||||||||||||||||||||||||||||||||||||||||||||||||||||||||||
Sbjct  1561  CACAGAACCGATTCCAGGAGGACAGCCCACACCTCCAGCTACCCCTGACTCGCCGGGACT  1620

Query  6851  TCCTTTTGCAGCAACAACTTTTGGGCGGAGACATTTGGCCACATCAGACGGGGGCGCAAG  6910
             ||||||||||||||||||||||||||||||||||||||||||||||||||||||||||||
Sbjct  1621  TCCTTTTGCAGCAACAACTTTTGGGCGGAGACATTTGGCCACATCAGACGGGGGCGCAAG  1680

Query  6911  TGAACCAAAGAGGTACTGCCCTCATCCTGACACTGTCAGCTGCGACAGCCACAGAGGGGG  6970
             ||||||||||||||||||||||||||||||||||||||||||||||||||||||||||||
Sbjct  1681  TGAACCAAAGAGGTACTGCCCTCATCCTGACACTGTCAGCTGCGACAGCCACAGAGGGGG  1740

Query  6971  AGAGGGCCAGTGTACCCTCTCCGGGACCACGGGACGAAGCCCAGGGCTACTATTGCATCC  7030
             ||||||||||||||||||||||||||||||||||||||||||||||||||||||||||||
Sbjct  1741  AGAGGGCCAGTGTACCCTCTCCGGGACCACGGGACGAAGCCCAGGGCTACTATTGCATCC  1800

Query  7031  AccccccgccccccGCCTGCAGGCGGCGGATGAGGCGGACCCGGCACAGACAGAGGCAGC  7090
             ||||||||||||||||||||||||||||||||||||||||||||||||||||||||||||
Sbjct  1801  ACCCCCCGCCCCCCGCCTGCAGGCGGCGGATGAGGCGGACCCGGCACAGACAGAGGCAGC  1860

Query  7091  AGCTCCGTCGGACCCTGGCAGACCTAAGGCAACAGCTTCAGGAGCAGGGGCTTCTCCTTC  7150
             ||||||||||||||||||||||||||||||||||||||||||||||||||||||||||||
Sbjct  1861  AGCTCCGTCGGACCCTGGCAGACCTAAGGCAACAGCTTCAGGAGCAGGGGCTTCTCCTTC  1920

Query  7151  GCATCGAGATACTGTAGGACCTCTCTTCCTATGCTTGTCAGGGGGCGCCAATAGCCTGAA  7210
             ||||||||||||||||||||||||||||||||||||||||||||||||||||||||||||
Sbjct  1921  GCATCGAGATACTGTAGGACCTCTCTTCCTATGCTTGTCAGGGGGCGCCAATAGCCTGAA  1980
```

```
Query  7211  GTGCCTGCGTTTCCGGTGCAAAAAGTACAAAGCCTCCTTGTTTGACTCTATGTCCACAAC  7270
             ||||||||||||||||||||||||||||||||||||||||||||||||||||||||||||
Sbjct  1981  GTGCCTGCGTTTCCGGTGCAAAAAGTACAAAGCCTCCTTGTTTGACTCTATGTCCACAAC  2040

Query  7271  ATTCTTTTGGACGGGACCTGAGGGCAAGGAGAGAGTGGGTGAAGCCCGTGTGCTGGTTAC  7330
             ||||||||||||||||||||||||||||||||||||||||||||||||||||||||||||
Sbjct  2041  ATTCTTTTGGACGGGACCTGAGGGCAAGGAGAGAGTGGGTGAAGCCCGTGTGCTGGTTAC  2100

Query  7331  ATTCTCGGGGGCACAGCAGAAGCAGGACTTCATGGAGAAGGTCTCACTGCCCCCCGGTGT  7390
             ||||||||||||||||||||||||||||||||||||||||||||||||||||||||||||
Sbjct  2101  ATTCTCGGGGGCACAGCAGAAGCAGGACTTCATGGAGAAGGTCTCACTGCCCCCCGGTGT  2160

Query  7391  GCGGGCGAGCCAGGCTGTCATGTGACTGTGGACATGGTACTGGACCTTGTGCATGCTGCT  7450
             ||||||||||||||||||||||||||||||||||||||||||||||||||||||||||||
Sbjct  2161  GCGGGCGAGCCAGGCTGTCATGTGACTGTGGACATGGTACTGGACCTTGTGCATGCTGCT  2220

Query  7451  GTTTTGATTTTGTGCCTTGTTGTGTTGTTGGAGTATTGCTTGGtttttttACTTGTTATT  7510
             ||||||||||||||||||||||||||||||||||||||||||||||||||||||||||||
Sbjct  2221  GTTTTGATTTTGTGCCTTGTTGTGTTGTTGGAGTATTGCTTGGTTTTTTTACTTGTTATT  2280

Query  7511  TTAGTAAATTTTCACTAAAACCTGTGTCATGGCGCTGCTTGccccccccccAGGGAAGCG  7570
             ||||||||||||||||||||||||||||||||||||||||||||||||||||||||||||
Sbjct  2281  TTAGTAAATTTTCACTAAAACCTGTGTCATGGCGCTGCTTGCCCCCCCCCCAGGGAAGCG  2340

Query  7571  GCGGCGCCGCGCCGCGGAGGGTGACCTGTACCGAGGG  7607
             |||||||||||||||||||||||||||||||||||||
Sbjct  2341  GCGGCGCCGCGCCGCGGAGGGTGACCTGTACCGAGGG  2377

################################################################################
```

RID: NR7Z2D2J114
Job Title:NODE_467_length_7607_cov_16.015890
Program: BLASTN
Query: NODE_467_length_7607_cov_16.015890 ID: lcl|Query_1211(dna) Length: 7607
Subject:NODE_2291_length_1856_cov_4.902192 ID: lcl|Query_1213(dna) Length: 1856

Sequences producing significant alignments:

```
                                                                    Scientific
Common                     Max    Total Query   E   Per.   Acc.
Description                                                         Name
Name            Taxid      Score  Score cover Value Ident  Len          Accession
NODE_2291_length_1856_cov_4.902192
0          3422   3422  24%   0.0   99.95  1856       Query_1213
```

Alignments:

>NODE_2291_length_1856_cov_4.902192
Sequence ID: Query_1213 Length: 1856
Range 1: 1 to 1856

Score:3422 bits(1853), Expect:0.0,
Identities:1855/1856(99%), Gaps:0/1856(0%), Strand: Plus/Plus

```
Query  3303  GGGCTTCCGAAACAATGCTGGCACGGAATCTATGGCACAGACCGCCGCGCGTTCTGCCAA  3362
             ||||||||||||||||||||||||||||||||||||||||||||||||||||||||||||
Sbjct  1     GGGCTTCCGAAACAATGCTGGCACGGAATCTATGGCACAGACCGCCGCGCGTTCTGCCAA  60

Query  3363  GTTCAGGCGGGCCCAAGCATGACTGTTGGTAGAAAGACCGCCAGCGGTTCTGGTCACCGC  3422
             ||||||||||||||||||||||||||||||||||||||||||||||||||||||||||||
Sbjct  61    GTTCAGGCGGGCCCAAGCATGACTGTTGGTAGAAAGACCGCCAGCGGTTCTGGTCACCGC  120

Query  3423  AGGCAGCCAACCGCGCGCGGTCCTCGGAGGCGCCACTTTCAAATTCTGGCGCCAAGCAGA  3482
             ||||||||||||||||||||||||||||||||||||||||||||||||||||||||||||
Sbjct  121   AGGCAGCCAACCGCGCGCGGTCCTCGGAGGCGCCACTTTCAAATTCTGGCGCCAAGCAGA  180
```

```
Query  3483  AAGAGGGTGTTCCCAACGGTTCTTTCTTGGCACAGGCAGCAACCTCGACCGCGAAAGGTC  3542
             ||||||||||||||||||||||||||||||||||||||||||||||||||||||||||||
Sbjct  181   AAGAGGGTGTTCCCAACGGTTCTTTCTTGGCACAGGCAGCAACCTCGACCGCGAAAGGTC  240

Query  3543  TAATAGGGGCGTGATTTTGGACCGTGGTCGGTCTCTGCAAGTTATTCTTTTCATGATGAT  3602
             ||||||||||||||||||||||||||||||||||||||||||||||||||||||||||||
Sbjct  241   TAATAGGGGCGTGATTTTGGACCGTGGTCGGTCTCTGCAAGTTATTCTTTTCATGATGAT  300

Query  3603  TGTTGTTAACAATAATATGAAAGATCAGCATGTTCCACGCACCGCCTGCGCCACCCTCAG  3662
             ||||||||||||||||||||||||||||||||||||||||||||||||||||||||||||
Sbjct  301   TGTTGTTAACAATAATATGAAAGATCAGCATGTTCCACGCACCGCCTGCGCCACCCTCAG  360

Query  3663  TCCCAGGAGGGGGGATAGACAGTTACACACCAGATGGAGATAGCTGTGCCTGAAAAGCAG  3722
             ||||||||||||||||||||||||||||||||||||||||||||||||||||||||||||
Sbjct  361   TCCCAGGAGGGGGGATAGACAGTTACACACCAGATGGAGATAGCTGTGCCTGAAAAGCAG  420

Query  3723  ACAGTAGGCCTGCCCTGTGTCTTTTGTGGACTGGCCATTGGACATTGGGACGCCCACTGG  3782
             ||||||||||||||||||||||||||||||||||||||||||||||||||||||||||||
Sbjct  421   ACAGTAGGCCTGCCCTGTGTCTTTTGTGGACTGGCCATTGGACATTGGGACGCCCACTGG  480

Query  3783  TTCGAGCATGGCGTGTACCAGCTGAGCTTCAAGGACGGGAAGCCCCACGCTGCACACCGT  3842
             ||||||||||||||||||||||||||||||||||||||||||||||||||||||||||||
Sbjct  481   TTCGAGCATGGCGTGTACCAGCTGAGCTTCAAGGACGGGAAGCCCCACGCTGCACACCGT  540

Query  3843  GATTGCCTTAAGGAGGCGTGCAAGAGAGAGCACGAGAGGCACAGAGACCCCAAGGTGTAC  3902
             ||||||||||||||||||||||||||||||||||||||||||||||||||||||||||||
Sbjct  541   GATTGCCTTAAGGAGGCGTGCAAGAGAGAGCACGAGAGGCACAGAGACCCCAAGGTGTAC  600

Query  3903  ACTGGCACTGAGGTGGAGGACACAGTCGGCCTGAAGGTCAAGTGGATCAGCGTGCGCTGC  3962
             ||||||||||||||||||||||||||||||||||||||||||||||||||||||||||||
Sbjct  601   ACTGGCACTGAGGTGGAGGACACAGTCGGCCTGAAGGTCAAGTGGATCAGCGTGCGCTGC  660

Query  3963  CACGTCTGCGGCTACCTGCTTACAGAAGACGACAAAGAGTACTGCAAGCTGTGCAGCTTC  4022
             ||||||||||||||||||||||||||||||||||||||||||||||||||||||||||||
Sbjct  661   CACGTCTGCGGCTACCTGCTTACAGAAGACGACAAAGAGTACTGCAAGCTGTGCAGCTTC  720

Query  4023  CCCTTCTACCATACACGCGGCCGCTGGCGCACCAGGTGCTACCACTGCAGGGTAGGCGAT  4082
             ||||||||||||||||||||||||||||||||||||||||||||||||||||||||||||
Sbjct  721   CCCTTCTACCATACACGCGGCCGCTGGCGCACCAGGTGCTACCACTGCAGGGTAGGCGAT  780

Query  4083  GAGGAGGATTGAGAGCGACCAAGAAGACAGTGGTGTAGAGTCCGACTTCGAGGAGGAGCA  4142
             ||||||||||||||||||||||||||||||||||||||||||||||||||||||||||||
Sbjct  781   GAGGAGGATTGAGAGCGACCAAGAAGACAGTGGTGTAGAGTCCGACTTCGAGGAGGAGCA  840

Query  4143  GGCAGATCGGGAGCCGGAAAGACTGGCTCCATACCGGATTGTGTTGCCCTGTGCGTGCTG  4202
             ||||||||||||||||||||||||||||||||||||||||||||||||||||||||||||
Sbjct  841   GGCAGATCGGGAGCCGGAAAGACTGGCTCCATACCGGATTGTGTTGCCCTGTGCGTGCTG  900

Query  4203  CGAGTGTCTCCTGCGCATGGTGGTACAGTGCAGCAGTACAGACATCAACGGTCTCAACCG  4262
             ||||||||||||||||||||||||||||||||||||||||||||||||||||||||||||
Sbjct  901   CGAGTGTCTCCTGCGCATGGTGGTACAGTGCAGCAGTACAGACATCAACGGTCTCAACCG  960

Query  4263  CCTGCTCTGCGGTTCCCTGGGCATCCTGTGTCCAACCTGTGCAGTGGAGAGAGGATACCA  4322
             ||||||||||||||||||||||||||||||||||||||||||||||||||||||||||||
Sbjct  961   CCTGCTCTGCGGTTCCCTGGGCATCCTGTGTCCAACCTGTGCAGTGGAGAGAGGATACCA  1020

Query  4323  TGGCCGATGAACCAGGTACCCCCGGGGAAGGCACTAGCGGGGCCTGGTTTCTTGCCAGGG  4382
             ||||||||||||||||||||||||||||||||||||||||||||||||||||||||||||
Sbjct  1021  TGGCCGATGAACCAGGTACCCCCGGGGAAGGCACTAGCGGGGCCTGGTTTCTTGCCAGGG  1080

Query  4383  AAGCGGTATGTAGTGGGTCAGATAGCGACGAAGAGGAGGCGGCTGATGTGTATGAAGGCC  4442
             ||||||||||||||||||||||||||||||||||||||||||||||||||||||||||||
Sbjct  1081  AAGCGGTATGTAGTGGGTCAGATAGCGACGAAGAGGAGGCGGCTGATGTGTATGAAGGCC  1140
```

```
Query  4443  CAGACCTAGACTTTGTAGACAATGCCTCCTATCACCAGGGAAATTCCCTGTACCTCCTCC  4502
             ||||||||||||||||||||||||||||||||||||||||||||||||||||||||||||
Sbjct  1141  CAGACCTAGACTTTGTAGACAATGCCTCCTATCACCAGGGAAATTCCCTGTACCTCCTCC  1200

Query  4503  AACAGCAAGAAGCGGAGGAGGACCGGCTGCATGTGCAGCTGCTAAAACGAAAATATGTAG  4562
             ||||||||||||||||||||||||||||||||||||||||||||||||||||||||||||
Sbjct  1201  AACAGCAAGAAGCGGAGGAGGACCGGCTGCATGTGCAGCTGCTAAAACGAAAATATGTAG  1260

Query  4563  AGAGCCCTAAACAGCGATTATGCCTGGCGCTCAGCCCCAGGCTGCAGGCTATTCAAATTT  4622
             ||||||||||||||||||||||||||||||||||||||||||||||||||||||||||||
Sbjct  1261  AGAGCCCTAAACAGCGATTATGCCTGGCGCTCAGCCCCAGGCTGCAGGCTATTCAAATTT  1320

Query  4623  CCCCCAATAAACAGCCTGTGAAAAGGCGCCTGTTTCAGCATCAGGACCAGGACAGCGGTC  4682
             ||||||||||||||||||||||||||||||||||||||||||||||||||||||||||||
Sbjct  1321  CCCCCAATAAACAGCCTGTGAAAAGGCGCCTGTTTCAGCATCAGGACCAGGACAGCGGTC  1380

Query  4683  TGGACCTGTCTCTGCAAAATGAAACTGCTAGTAATGACCAGGACGGGACCCACATTCAGG  4742
             ||||||||||||||||||||||||||||||||||||||||||||||||||||||||||||
Sbjct  1381  TGGACCTGTCTCTGCAAAATGAAACTGCTAGTAATGACCAGGACGGGACCCACATTCAGG  1440

Query  4743  TAGACCCATGCGAAACTGTGGGGGACAGGAATGGAAATGGCGAAgggggggCGCGAGCCG  4802
             ||||||||||||||||||||||||||||||||||||||||||||||||||||||||||||
Sbjct  1441  TAGACCCATGCGAAACTGTGGGGGACAGGAATGGAAATGGCGAAGGGGGGGCGCGAGCCG  1500

Query  4803  GGGAGACCCCCTGGCAGCCAAGTAAGGCCGCCAGTGGGACAGAGGCACAGCCGCATCTGG  4862
             ||||||||||||||||||||||||||||||||||||||||||||||||||||||||||||
Sbjct  1501  GGGAGACCCCCTGGCAGCCAAGTAAGGCCGCCAGTGGGACAGAGGCACAGCCGCATCTGG  1560

Query  4863  ACGTACTCAGAGCCAGTAATCGGAGGGCGGCCATGTTGGCGCGCTTTAAAGAAGGCTTCG  4922
             ||||||||||||||||||||||||||||||||||||||| ||||||||||||||||||||
Sbjct  1561  ACGTACTCAGAGCCAGTAATCGGAGGGCGGCCATGTTGGTGCGCTTTAAAGAAGGCTTCG  1620

Query  4923  GTGTCAGCTTTTCTGAGCTCACGCGCAGCTTTAAAAGCGACCGCTCCTGTGTGGGGGACT  4982
             ||||||||||||||||||||||||||||||||||||||||||||||||||||||||||||
Sbjct  1621  GTGTCAGCTTTTCTGAGCTCACGCGCAGCTTTAAAAGCGACCGCTCCTGTGTGGGGGACT  1680

Query  4983  GGGTCGTGCTGGCGTTTGGCGTCCGAGAACAGTTTGCCGAAACCGCCAAAGAGCAGCTGA  5042
             ||||||||||||||||||||||||||||||||||||||||||||||||||||||||||||
Sbjct  1681  GGGTCGTGCTGGCGTTTGGCGTCCGAGAACAGTTTGCCGAAACCGCCAAAGAGCAGCTGA  1740

Query  5043  AGGGACACTGTGGATACGTGCAGTTCACGTATCGACCGGACGCTAGGGGTGCGCTTACAC  5102
             ||||||||||||||||||||||||||||||||||||||||||||||||||||||||||||
Sbjct  1741  AGGGACACTGTGGATACGTGCAGTTCACGTATCGACCGGACGCTAGGGGTGCGCTTACAC  1800

Query  5103  TAGCGCTGCTGTCATTTACCTGTCAAAAGAATAGGGACACTGTACGTAACCTGATG  5158
             ||||||||||||||||||||||||||||||||||||||||||||||||||||||||
Sbjct  1801  TAGCGCTGCTGTCATTTACCTGTCAAAAGAATAGGGACACTGTACGTAACCTGATG  1856
```

##
##
RID: NR7S1RCS114
Job Title:NODE_183_length_7543_cov_4.284892
Program: BLASTN
Query: NODE_183_length_7543_cov_4.284892 ID: lcl|Query_28745(dna) Length: 7543
Subject:NODE_467_length_7607_cov_16.015890 ID: lcl|Query_28747(dna) Length: 7607

Sequences producing significant alignments:

Description	Common Name	Taxid	Max Score	Total Score	Query cover	E Value	Per. Ident	Acc. Len	Scientific Name	Accession
NODE_467_length_7607_cov_16.015890	0		10770	14033	100%	0.0	100.00	7607		Query_28747

Alignments:

```
>NODE_467_length_7607_cov_16.015890
Sequence ID: Query_28747 Length: 7607
Range 1: 1 to 5832

Score:10770 bits(5832), Expect:0.0,
Identities:5832/5832(100%),  Gaps:0/5832(0%), Strand: Plus/Plus

Query  1712  cccccccccAGGGAAGCGGCGGCGCCGCGCCGCGGAGGGTGACCTGTACCGAGGGTGCAT  1771
             ||||||||||||||||||||||||||||||||||||||||||||||||||||||||||||
Sbjct  1     CCCCCCCCCAGGGAAGCGGCGGCGCCGCGCCGCGGAGGGTGACCTGTACCGAGGGTGCAT  60

Query  1772  TATGGGGCAGGACTGCCCGCCGGACATCAAGGCAAAGTTTGAGAACGACACACTTGCAGA  1831
             ||||||||||||||||||||||||||||||||||||||||||||||||||||||||||||
Sbjct  61    TATGGGGCAGGACTGCCCGCCGGACATCAAGGCAAAGTTTGAGAACGACACACTTGCAGA  120

Query  1832  CCGCTTTCTAAAGTGGGTCAGCTCCTTCCTCTACCTAGGCAACCTGGGTTTCAGCACGGG  1891
             ||||||||||||||||||||||||||||||||||||||||||||||||||||||||||||
Sbjct  121   CCGCTTTCTAAAGTGGGTCAGCTCCTTCCTCTACCTAGGCAACCTGGGTTTCAGCACGGG  180

Query  1892  CAGGGGCAATCCCAGGTTTGGGTACAGACCTGTggggggcggggtaggcgaggcgggtgg  1951
             ||||||||||||||||||||||||||||||||||||||||||||||||||||||||||||
Sbjct  181   CAGGGGCAATCCCAGGTTTGGGTACAGACCTGTGGGGGGCGGGGTAGGCGAGGCGGGTGG  240

Query  1952  ggggggtgcgggtcgggACGCCCATTACGGACGTGTCCCCGGGGGTCACTGTGGAGACGGT  2011
             ||||||||||||||||||||||||||||||||||||||||||||||||||||||||||||
Sbjct  241   GGGGGTGCGGGTCGGGACGCCCATTACGGACGTGTCCCCGGGGGTCACTGTGGAGACGGT  300

Query  2012  GGGGCCCGCGGAGGTGGTCCCAGTAGACAGTTTAAGCCCCTCAGCCCCGGCTGTGGTGCC  2071
             ||||||||||||||||||||||||||||||||||||||||||||||||||||||||||||
Sbjct  301   GGGGCCCGCGGAGGTGGTCCCAGTAGACAGTTTAAGCCCCTCAGCCCCGGCTGTGGTGCC  360

Query  2072  TGCGGGGGAAGGCACAGGGGGCGCTGTGGATGTGGAGTTGGTGGCTGAGGTGCACCCTAC  2131
             ||||||||||||||||||||||||||||||||||||||||||||||||||||||||||||
Sbjct  361   TGCGGGGGAAGGCACAGGGGGCGCTGTGGATGTGGAGTTGGTGGCTGAGGTGCACCCTAC  420

Query  2132  CGTGGACCCGGGCATGGCTGGGGGCTCGGGGGGTGTTACCAGCAACGGCGGGGATGCCGC  2191
             ||||||||||||||||||||||||||||||||||||||||||||||||||||||||||||
Sbjct  421   CGTGGACCCGGGCATGGCTGGGGGCTCGGGGGGTGTTACCAGCAACGGCGGGGATGCCGC  480

Query  2192  CGTGCTGGAGGTGCCCCCTGAGGTGGTTCCCCGGGGTCGGAATGCTACTAGCCGCACACA  2251
             ||||||||||||||||||||||||||||||||||||||||||||||||||||||||||||
Sbjct  481   CGTGCTGGAGGTGCCCCCTGAGGTGGTTCCCCGGGGTCGGAATGCTACTAGCCGCACACA  540

Query  2252  GTTTCATAATCCTGCATTCCATGTGGAGCTAAACAGCTCGCTCCCCACGGGTGAATCGTC  2311
             ||||||||||||||||||||||||||||||||||||||||||||||||||||||||||||
Sbjct  541   GTTTCATAATCCTGCATTCCATGTGGAGCTAAACAGCTCGCTCCCCACGGGTGAATCGTC  600

Query  2312  CGCCTCTGACCATGTGTTTGTCCAGGCGGAACAGGGTGGTCACTGGGTGGGAGAGGAAAT  2371
             ||||||||||||||||||||||||||||||||||||||||||||||||||||||||||||
Sbjct  601   CGCCTCTGACCATGTGTTTGTCCAGGCGGAACAGGGTGGTCACTGGGTGGGAGAGGAAAT  660

Query  2372  TGAATTGCTTCCTTTGGGCGATACGCTGACGCAAAGAACCAGCACCCCCAGAACTGGGGC  2431
             ||||||||||||||||||||||||||||||||||||||||||||||||||||||||||||
Sbjct  661   TGAATTGCTTCCTTTGGGCGATACGCTGACGCAAAGAACCAGCACCCCCAGAACTGGGGC  720

Query  2432  ACGGGGACGCGCAGAGAGCGGGGCGGAGCGTCTATTTGGACGTGGCTTCCGTCAGGTGCG  2491
             ||||||||||||||||||||||||||||||||||||||||||||||||||||||||||||
Sbjct  721   ACGGGGACGCGCAGAGAGCGGGGCGGAGCGTCTATTTGGACGTGGCTTCCGTCAGGTGCG  780

Query  2492  CGTAACCGATCCAAACTTCCTCTCCGACCCTGGGTCTCTTGTGCAGTTTGGGTTCGAGAA  2551
             ||||||||||||||||||||||||||||||||||||||||||||||||||||||||||||
```

```
Sbjct  781   CGTAACCGATCCAAACTTCCTCTCCGACCCTGGGTCTCTTGTGCAGTTTGGGTTCGAGAA  840

Query  2552  CCCTGCATATGACCCCTCCGGCAGCATCTCCTTTGGGCCACTCGGCGAGCCGGAGGCGGC  2611
             ||||||||||||||||||||||||||||||||||||||||||||||||||||||||||||
Sbjct  841   CCCTGCATATGACCCCTCCGGCAGCATCTCCTTTGGGCCACTCGGCGAGCCGGAGGCGGC  900

Query  2612  CCCCAACCCAGAGTTTCAGGATGTTGTGCACCTTGGCAGGACACACATTACAGAGCGCGA  2671
             ||||||||||||||||||||||||||||||||||||||||||||||||||||||||||||
Sbjct  901   CCCCAACCCAGAGTTTCAGGATGTTGTGCACCTTGGCAGGACACACATTACAGAGCGCGA  960

Query  2672  CGGGCGCGTGGTGCTAGGAAGATTTGGTCAGCGGGCTGGGGTGTCCACCCGCAGCGGGCG  2731
             ||||||||||||||||||||||||||||||||||||||||||||||||||||||||||||
Sbjct  961   CGGGCGCGTGGTGCTAGGAAGATTTGGTCAGCGGGCTGGGGTGTCCACCCGCAGCGGGCG  1020

Query  2732  GGTGGTGGGACCCCAGGTACACTATAGGTTTGAGTTCAGCAGCATTGCCCCTGCAGAGGA  2791
             ||||||||||||||||||||||||||||||||||||||||||||||||||||||||||||
Sbjct  1021  GGTGGTGGGACCCCAGGTACACTATAGGTTTGAGTTCAGCAGCATTGCCCCTGCAGAGGA  1080

Query  2792  GATTGAACTGGTGCCTCTGCCAAGTGCGGATCCGCCGGGGTCACTTGGGGACGAGTTTGA  2851
             ||||||||||||||||||||||||||||||||||||||||||||||||||||||||||||
Sbjct  1081  GATTGAACTGGTGCCTCTGCCAAGTGCGGATCCGCCGGGGTCACTTGGGGACGAGTTTGA  1140

Query  2852  GGTTATCGACCTGGACAGCACAGGTAGTGTGTACAGCGAGGCGGCTCTGCTGGACGATGA  2911
             ||||||||||||||||||||||||||||||||||||||||||||||||||||||||||||
Sbjct  1141  GGTTATCGACCTGGACAGCACAGGTAGTGTGTACAGCGAGGCGGCTCTGCTGGACGATGA  1200

Query  2912  CAGCCTCAGCATTCACGGGGTGCTTAGCTTGGGTCAGAGAGCTGGCAGCCACCGCGCTGT  2971
             ||||||||||||||||||||||||||||||||||||||||||||||||||||||||||||
Sbjct  1201  CAGCCTCAGCATTCACGGGGTGCTTAGCTTGGGTCAGAGAGCTGGCAGCCACCGCGCTGT  1260

Query  2972  GTCCGTCCTGGACTTTGCTGGACCTAGAGCCTTTTACAGTGCACTGACAGTAGAGGGCGA  3031
             ||||||||||||||||||||||||||||||||||||||||||||||||||||||||||||
Sbjct  1261  GTCCGTCCTGGACTTTGCTGGACCTAGAGCCTTTTACAGTGCACTGACAGTAGAGGGCGA  1320

Query  3032  CGTGGCACCTGCACACCCGAACCCCACTAGCTCTATCCTTGTGGTCCCTGAGAACGCCGA  3091
             ||||||||||||||||||||||||||||||||||||||||||||||||||||||||||||
Sbjct  1321  CGTGGCACCTGCACACCCGAACCCCACTAGCTCTATCCTTGTGGTCCCTGAGAACGCCGA  1380

Query  3092  GCAGACCCCCTCTGTGGTTGTTGAATCGATGCCGGTCAGTGGCCTGTATGACCTGCACCC  3151
             ||||||||||||||||||||||||||||||||||||||||||||||||||||||||||||
Sbjct  1381  GCAGACCCCCTCTGTGGTTGTTGAATCGATGCCGGTCAGTGGCCTGTATGACCTGCACCC  1440

Query  3152  CAGCTTGCACCCTAGACGCAGGCGCAAACGCAGGCGTTCCTTCTGTTTTGCAGATGGCAG  3211
             ||||||||||||||||||||||||||||||||||||||||||||||||||||||||||||
Sbjct  1441  CAGCTTGCACCCTAGACGCAGGCGCAAACGCAGGCGTTCCTTCTGTTTTGCAGATGGCAG  1500

Query  3212  TGTGGACACAAAACAAGCAGAAGCTTTTTCTGCCTCCGCCGCCTGTTACTAGAGTCCCTA  3271
             ||||||||||||||||||||||||||||||||||||||||||||||||||||||||||||
Sbjct  1501  TGTGGACACAAAACAAGCAGAAGCTTTTTCTGCCTCCGCCGCCTGTTACTAGAGTCCCTA  1560

Query  3272  ACACAGATGAGTATGTCGTGCGACACAACATATTTTACCATGGTAACAGTGACCGACTGC  3331
             ||||||||||||||||||||||||||||||||||||||||||||||||||||||||||||
Sbjct  1561  ACACAGATGAGTATGTCGTGCGACACAACATATTTTACCATGGTAACAGTGACCGACTGC  1620

Query  3332  TTATGGTCGGCCACCCTTTCTTTCCTGTGAAGAGTGGCAACATAGATGTTCCTAAGGTGT  3391
             ||||||||||||||||||||||||||||||||||||||||||||||||||||||||||||
Sbjct  1621  TTATGGTCGGCCACCCTTTCTTTCCTGTGAAGAGTGGCAACATAGATGTTCCTAAGGTGT  1680

Query  3392  CTGCAAATCAATTCAGGGTCTTTAAGGTGCTTCTGCCTGACCCTAATAAGTTTGCTCTGG  3451
             ||||||||||||||||||||||||||||||||||||||||||||||||||||||||||||
Sbjct  1681  CTGCAAATCAATTCAGGGTCTTTAAGGTGCTTCTGCCTGACCCTAATAAGTTTGCTCTGG  1740

Query  3452  CCGACCCTGACTTATACAACCCGGAGACGGGCCGCCTGGTATGGGCCCTAAAGGGCTTGG  3511
             ||||||||||||||||||||||||||||||||||||||||||||||||||||||||||||
```

```
Sbjct  1741  CCGACCCTGACTTATACAACCCGGAGACGGGCCGCCTGGTATGGGCCCTAAAGGGCTTGG  1800

Query  3512  AGGTTAGCAGGGGCCAGCCCCTGGGCATAGGTGTCACAGGTAACCCCCTGTTTAACAAAA  3571
             ||||||||||||||||||||||||||||||||||||||||||||||||||||||||||||
Sbjct  1801  AGGTTAGCAGGGGCCAGCCCCTGGGCATAGGTGTCACAGGTAACCCCCTGTTTAACAAAA  1860

Query  3572  AGAATGATGTTGAAAACCCTAGCAAGCTACAGGGGGCAGGTGCCAAGGATGACAGGGTGA  3631
             ||||||||||||||||||||||||||||||||||||||||||||||||||||||||||||
Sbjct  1861  AGAATGATGTTGAAAACCCTAGCAAGCTACAGGGGGCAGGTGCCAAGGATGACAGGGTGA  1920

Query  3632  ACATGGGCTTTGATGTGAAGCAACACCAGCTGCTGCTGGTGGGTTGCAAGCCCCCACAGG  3691
             ||||||||||||||||||||||||||||||||||||||||||||||||||||||||||||
Sbjct  1921  ACATGGGCTTTGATGTGAAGCAACACCAGCTGCTGCTGGTGGGTTGCAAGCCCCCACAGG  1980

Query  3692  GGGAGCACTGGAGGAAGACACGCTTTTGCAACACAGGACAGCCACCGGTGGGCTCCTGCC  3751
             ||||||||||||||||||||||||||||||||||||||||||||||||||||||||||||
Sbjct  1981  GGGAGCACTGGAGGAAGACACGCTTTTGCAACACAGGACAGCCACCGGTGGGCTCCTGCC  2040

Query  3752  CGGCCATAGAGCTGGTCAATACCGTGATAGAGGATGGGGACATGGGGGACGTGGGTTTCG  3811
             ||||||||||||||||||||||||||||||||||||||||||||||||||||||||||||
Sbjct  2041  CGGCCATAGAGCTGGTCAATACCGTGATAGAGGATGGGGACATGGGGGACGTGGGTTTCG  2100

Query  3812  GGGCAATGGACTTTGCCACACTTTGTGACAGTAAGGCCGATGTGCCTCTGGACCTCGTGG  3871
             ||||||||||||||||||||||||||||||||||||||||||||||||||||||||||||
Sbjct  2101  GGGCAATGGACTTTGCCACACTTTGTGACAGTAAGGCCGATGTGCCTCTGGACCTCGTGG  2160

Query  3872  GCACTGCCTCGAAGTACCCCGACTACATTAAGATGGGGCAGGAGCCTGCAGGGGACAGCA  3931
             ||||||||||||||||||||||||||||||||||||||||||||||||||||||||||||
Sbjct  2161  GCACTGCCTCGAAGTACCCCGACTACATTAAGATGGGGCAGGAGCCTGCAGGGGACAGCA  2220

Query  3932  TGTGGTTTTTCGCCAGGAGGGAGCAGTACTATGCCAGGCACTTTTTTACGCGGGACGGTA  3991
             ||||||||||||||||||||||||||||||||||||||||||||||||||||||||||||
Sbjct  2221  TGTGGTTTTTCGCCAGGAGGGAGCAGTACTATGCCAGGCACTTTTTTACGCGGGACGGTA  2280

Query  3992  AGAGTCTGGAGACGGTGCCCCCCGAGTTGTACACAGCACCAGAGGCAAACCCAACCAACA  4051
             ||||||||||||||||||||||||||||||||||||||||||||||||||||||||||||
Sbjct  2281  AGAGTCTGGAGACGGTGCCCCCCGAGTTGTACACAGCACCAGAGGCAAACCCAACCAACA  2340

Query  4052  TCAACAGGTACATTTACAGTGCAAGCCCCAGTGGGTCTCTCGTGTCCACAGACTCACAGA  4111
             ||||||||||||||||||||||||||||||||||||||||||||||||||||||||||||
Sbjct  2341  TCAACAGGTACATTTACAGTGCAAGCCCCAGTGGGTCTCTCGTGTCCACAGACTCACAGA  2400

Query  4112  TATTTAACAGGCCGTACTGGCTGGCACGCGCACAGGGCCAGAATAATGGCATCTGCTGGC  4171
             ||||||||||||||||||||||||||||||||||||||||||||||||||||||||||||
Sbjct  2401  TATTTAACAGGCCGTACTGGCTGGCACGCGCACAGGGCCAGAATAATGGCATCTGCTGGC  2460

Query  4172  ACAACACTTTGTATGTGACCGTGTTTGACAACACACGTGGCACAAACTTGACCATTACAG  4231
             ||||||||||||||||||||||||||||||||||||||||||||||||||||||||||||
Sbjct  2461  ACAACACTTTGTATGTGACCGTGTTTGACAACACACGTGGCACAAACTTGACCATTACAG  2520

Query  4232  TGAACCCCCAGAACACATATGATTCCACAGGCTTTAATGCATACGTGCGACATGTGGAGG  4291
             ||||||||||||||||||||||||||||||||||||||||||||||||||||||||||||
Sbjct  2521  TGAACCCCCAGAACACATATGATTCCACAGGCTTTAATGCATACGTGCGACATGTGGAGG  2580

Query  4292  AGTTTGAGCTCTCCTTTATCTTTCAGCTGTGCACGGTCCCCTTGACACCCCAGGTCCTTG  4351
             ||||||||||||||||||||||||||||||||||||||||||||||||||||||||||||
Sbjct  2581  AGTTTGAGCTCTCCTTTATCTTTCAGCTGTGCACGGTCCCCTTGACACCCCAGGTCCTTG  2640

Query  4352  CCCACCTCCATACAACCGATGCCAGCCTCCTTGAGGAGTGGGAGATTGGGGTAAAccccc  4411
             ||||||||||||||||||||||||||||||||||||||||||||||||||||||||||||
Sbjct  2641  CCCACCTCCATACAACCGATGCCAGCCTCCTTGAGGAGTGGGAGATTGGGGTAAACCCCC  2700

Query  4412  ccTCCTCCTCGCAGCTGGAGGACACATACCGGTTTATTACCTCTACTGCCACAAAGTGCC  4471
             ||||||||||||||||||||||||||||||||||||||||||||||||||||||||||||
```

```
Sbjct  2701  CCTCCTCCTCGCAGCTGGAGGACACATACCGGTTTATTACCTCTACTGCCACAAAGTGCC  2760

Query  4472  CTGTGCCTCCCCAGGCCCCGCTTGAGCCACCGGGGTACACATTTTGGACTGTGGATCTGC  4531
             ||||||||||||||||||||||||||||||||||||||||||||||||||||||||||||
Sbjct  2761  CTGTGCCTCCCCAGGCCCCGCTTGAGCCACCGGGGTACACATTTTGGACTGTGGATCTGC  2820

Query  4532  AGGAGCGTTTGTCCTTGGACCTGGACCAGTACACCCTGGGGAGGCGCTTTTTGGCGCAGT  4591
             ||||||||||||||||||||||||||||||||||||||||||||||||||||||||||||
Sbjct  2821  AGGAGCGTTTGTCCTTGGACCTGGACCAGTACACCCTGGGGAGGCGCTTTTTGGCGCAGT  2880

Query  4592  CAGGGGTGTCTACCACACGCTCGCTTAGGGCCCCCACCTCCAGAAAGCGCAAGTCATCAG  4651
             ||||||||||||||||||||||||||||||||||||||||||||||||||||||||||||
Sbjct  2881  CAGGGGTGTCTACCACACGCTCGCTTAGGGCCCCCACCTCCAGAAAGCGCAAGTCATCAG  2940

Query  4652  GGTCGGCCACCAGAAGGACCACCAAAAGAAGGAAATAGTGTGTATGCCATTCTGTTTTTC  4711
             ||||||||||||||||||||||||||||||||||||||||||||||||||||||||||||
Sbjct  2941  GGTCGGCCACCAGAAGGACCACCAAAAGAAGGAAATAGTGTGTATGCCATTCTGTTTTTC  3000

Query  4712  ATATTTAATAAAGACTTTTGAATAAGCACTGTGTCATTCATGTTCATGCACCGCACCCGG  4771
             ||||||||||||||||||||||||||||||||||||||||||||||||||||||||||||
Sbjct  3001  ATATTTAATAAAGACTTTTGAATAAGCACTGTGTCATTCATGTTCATGCACCGCACCCGG  3060

Query  4772  TGCAAGTATGCACCGCCTGGGATATATACCGTCCGCGGTGCCATCGCACCTGGTGCTCAC  4831
             ||||||||||||||||||||||||||||||||||||||||||||||||||||||||||||
Sbjct  3061  TGCAAGTATGCACCGCCTGGGATATATACCGTCCGCGGTGCCATCGCACCTGGTGCTCAC  3120

Query  4832  TTCGTGCCAGCTGTACCATTGCCATGGAGGCAGAGGATTAAGGTAAGTGCTTTTAAATCC  4891
             ||||||||||||||||||||||||||||||||||||||||||||||||||||||||||||
Sbjct  3121  TTCGTGCCAGCTGTACCATTGCCATGGAGGCAGAGGATTAAGGTAAGTGCTTTTAAATCC  3180

Query  4892  TATGtttttttttCTCCATTGTTTAACTGCACAGTTTGTTCTAATGGAACTCTAGGCGTA  4951
             ||||||||||||||||||||||||||||||||||||||||||||||||||||||||||||
Sbjct  3181  TATGTTTTTTTTTCTCCATTGTTTAACTGCACAGTTTGTTCTAATGGAACTCTAGGCGTA  3240

Query  4952  CTGCCAGGTGGAGAGAATGTGCTGCGCCAGCAGAATCGACCGGGCGCGGTACACCTACAC  5011
             ||||||||||||||||||||||||||||||||||||||||||||||||||||||||||||
Sbjct  3241  CTGCCAGGTGGAGAGAATGTGCTGCGCCAGCAGAATCGACCGGGCGCGGTACACCTACAC  3300

Query  5012  CTGGGCTTCCGAAACAATGCTGGCACGGAATCTATGGCACAGACCGCCGCGCGTTCTGCC  5071
             ||||||||||||||||||||||||||||||||||||||||||||||||||||||||||||
Sbjct  3301  CTGGGCTTCCGAAACAATGCTGGCACGGAATCTATGGCACAGACCGCCGCGCGTTCTGCC  3360

Query  5072  AAGTTCAGGCGGGCCCAAGCATGACTGTTGGTAGAAAGACCGCCAGCGGTTCTGGTCACC  5131
             ||||||||||||||||||||||||||||||||||||||||||||||||||||||||||||
Sbjct  3361  AAGTTCAGGCGGGCCCAAGCATGACTGTTGGTAGAAAGACCGCCAGCGGTTCTGGTCACC  3420

Query  5132  GCAGGCAGCCAACCGCGCGCGGTCCTCGGAGGCGCCACTTTCAAATTCTGGCGCCAAGCA  5191
             ||||||||||||||||||||||||||||||||||||||||||||||||||||||||||||
Sbjct  3421  GCAGGCAGCCAACCGCGCGCGGTCCTCGGAGGCGCCACTTTCAAATTCTGGCGCCAAGCA  3480

Query  5192  GAAAGAGGGTGTTCCCAACGGTTCTTTCTTGGCACAGGCAGCAACCTCGACCGCGAAAGG  5251
             ||||||||||||||||||||||||||||||||||||||||||||||||||||||||||||
Sbjct  3481  GAAAGAGGGTGTTCCCAACGGTTCTTTCTTGGCACAGGCAGCAACCTCGACCGCGAAAGG  3540

Query  5252  TCTAATAGGGGCGTGATTTTGGACCGTGGTCGGTCTCTGCAAGTTATTCTTTTCATGATG  5311
             ||||||||||||||||||||||||||||||||||||||||||||||||||||||||||||
Sbjct  3541  TCTAATAGGGGCGTGATTTTGGACCGTGGTCGGTCTCTGCAAGTTATTCTTTTCATGATG  3600

Query  5312  ATTGTTGTTAACAATAATATGAAAGATCAGCATGTTCCACGCACCGCCTGCGCCACCCTC  5371
             ||||||||||||||||||||||||||||||||||||||||||||||||||||||||||||
Sbjct  3601  ATTGTTGTTAACAATAATATGAAAGATCAGCATGTTCCACGCACCGCCTGCGCCACCCTC  3660

Query  5372  AGTCCCAGGAGGGGGGATAGACAGTTACACACCAGATGGAGATAGCTGTGCCTGAAAAGC  5431
             ||||||||||||||||||||||||||||||||||||||||||||||||||||||||||||
```

```
Sbjct  3661  AGTCCCAGGAGGGGGGATAGACAGTTACACACCAGATGGAGATAGCTGTGCCTGAAAAGC  3720

Query  5432  AGACAGTAGGCCTGCCCTGTGTCTTTTGTGGACTGGCCATTGGACATTGGGACGCCCACT  5491
             ||||||||||||||||||||||||||||||||||||||||||||||||||||||||||||
Sbjct  3721  AGACAGTAGGCCTGCCCTGTGTCTTTTGTGGACTGGCCATTGGACATTGGGACGCCCACT  3780

Query  5492  GGTTCGAGCATGGCGTGTACCAGCTGAGCTTCAAGGACGGGAAGCCCCACGCTGCACACC  5551
             ||||||||||||||||||||||||||||||||||||||||||||||||||||||||||||
Sbjct  3781  GGTTCGAGCATGGCGTGTACCAGCTGAGCTTCAAGGACGGGAAGCCCCACGCTGCACACC  3840

Query  5552  GTGATTGCCTTAAGGAGGCGTGCAAGAGAGAGCACGAGAGGCACAGAGACCCCAAGGTGT  5611
             ||||||||||||||||||||||||||||||||||||||||||||||||||||||||||||
Sbjct  3841  GTGATTGCCTTAAGGAGGCGTGCAAGAGAGAGCACGAGAGGCACAGAGACCCCAAGGTGT  3900

Query  5612  ACACTGGCACTGAGGTGGAGGACACAGTCGGCCTGAAGGTCAAGTGGATCAGCGTGCGCT  5671
             ||||||||||||||||||||||||||||||||||||||||||||||||||||||||||||
Sbjct  3901  ACACTGGCACTGAGGTGGAGGACACAGTCGGCCTGAAGGTCAAGTGGATCAGCGTGCGCT  3960

Query  5672  GCCACGTCTGCGGCTACCTGCTTACAGAAGACGACAAAGAGTACTGCAAGCTGTGCAGCT  5731
             ||||||||||||||||||||||||||||||||||||||||||||||||||||||||||||
Sbjct  3961  GCCACGTCTGCGGCTACCTGCTTACAGAAGACGACAAAGAGTACTGCAAGCTGTGCAGCT  4020

Query  5732  TCCCCTTCTACCATACACGCGGCCGCTGGCGCACCAGGTGCTACCACTGCAGGGTAGGCG  5791
             ||||||||||||||||||||||||||||||||||||||||||||||||||||||||||||
Sbjct  4021  TCCCCTTCTACCATACACGCGGCCGCTGGCGCACCAGGTGCTACCACTGCAGGGTAGGCG  4080

Query  5792  ATGAGGAGGATTGAGAGCGACCAAGAAGACAGTGGTGTAGAGTCCGACTTCGAGGAGGAG  5851
             ||||||||||||||||||||||||||||||||||||||||||||||||||||||||||||
Sbjct  4081  ATGAGGAGGATTGAGAGCGACCAAGAAGACAGTGGTGTAGAGTCCGACTTCGAGGAGGAG  4140

Query  5852  CAGGCAGATCGGGAGCCGGAAAGACTGGCTCCATACCGGATTGTGTTGCCCTGTGCGTGC  5911
             ||||||||||||||||||||||||||||||||||||||||||||||||||||||||||||
Sbjct  4141  CAGGCAGATCGGGAGCCGGAAAGACTGGCTCCATACCGGATTGTGTTGCCCTGTGCGTGC  4200

Query  5912  TGCGAGTGTCTCCTGCGCATGGTGGTACAGTGCAGCAGTACAGACATCAACGGTCTCAAC  5971
             ||||||||||||||||||||||||||||||||||||||||||||||||||||||||||||
Sbjct  4201  TGCGAGTGTCTCCTGCGCATGGTGGTACAGTGCAGCAGTACAGACATCAACGGTCTCAAC  4260

Query  5972  CGCCTGCTCTGCGGTTCCCTGGGCATCCTGTGTCCAACCTGTGCAGTGGAGAGAGGATAC  6031
             ||||||||||||||||||||||||||||||||||||||||||||||||||||||||||||
Sbjct  4261  CGCCTGCTCTGCGGTTCCCTGGGCATCCTGTGTCCAACCTGTGCAGTGGAGAGAGGATAC  4320

Query  6032  CATGGCCGATGAACCAGGTACCCCCGGGGAAGGCACTAGCGGGGCCTGGTTTCTTGCCAG  6091
             ||||||||||||||||||||||||||||||||||||||||||||||||||||||||||||
Sbjct  4321  CATGGCCGATGAACCAGGTACCCCCGGGGAAGGCACTAGCGGGGCCTGGTTTCTTGCCAG  4380

Query  6092  GGAAGCGGTATGTAGTGGGTCAGATAGCGACGAAGAGGAGGCGGCTGATGTGTATGAAGG  6151
             ||||||||||||||||||||||||||||||||||||||||||||||||||||||||||||
Sbjct  4381  GGAAGCGGTATGTAGTGGGTCAGATAGCGACGAAGAGGAGGCGGCTGATGTGTATGAAGG  4440

Query  6152  CCCAGACCTAGACTTTGTAGACAATGCCTCCTATCACCAGGGAAATTCCCTGTACCTCCT  6211
             ||||||||||||||||||||||||||||||||||||||||||||||||||||||||||||
Sbjct  4441  CCCAGACCTAGACTTTGTAGACAATGCCTCCTATCACCAGGGAAATTCCCTGTACCTCCT  4500

Query  6212  CCAACAGCAAGAAGCGGAGGAGGACCGGCTGCATGTGCAGCTGCTAAAACGAAAATATGT  6271
             ||||||||||||||||||||||||||||||||||||||||||||||||||||||||||||
Sbjct  4501  CCAACAGCAAGAAGCGGAGGAGGACCGGCTGCATGTGCAGCTGCTAAAACGAAAATATGT  4560

Query  6272  AGAGAGCCCTAAACAGCGATTATGCCTGGCGCTCAGCCCCAGGCTGCAGGCTATTCAAAT  6331
             ||||||||||||||||||||||||||||||||||||||||||||||||||||||||||||
Sbjct  4561  AGAGAGCCCTAAACAGCGATTATGCCTGGCGCTCAGCCCCAGGCTGCAGGCTATTCAAAT  4620

Query  6332  TTCCCCCAATAAACAGCCTGTGAAAAGGCGCCTGTTTCAGCATCAGGACCAGGACAGCGG  6391
             ||||||||||||||||||||||||||||||||||||||||||||||||||||||||||||
```

```
Sbjct  4621  TTCCCCCAATAAACAGCCTGTGAAAAGGCGCCTGTTTCAGCATCAGGACCAGGACAGCGG  4680

Query  6392  TCTGGACCTGTCTCTGCAAAATGAAACTGCTAGTAATGACCAGGACGGGACCCACATTCA  6451
             ||||||||||||||||||||||||||||||||||||||||||||||||||||||||||||
Sbjct  4681  TCTGGACCTGTCTCTGCAAAATGAAACTGCTAGTAATGACCAGGACGGGACCCACATTCA  4740

Query  6452  GGTAGACCCATGCGAAACTGTGGGGGACAGGAATGGAAATGGCGAAgggggggCGCGAGC  6511
             ||||||||||||||||||||||||||||||||||||||||||||||||||||||||||||
Sbjct  4741  GGTAGACCCATGCGAAACTGTGGGGGACAGGAATGGAAATGGCGAAGGGGGGGCGCGAGC  4800

Query  6512  CGGGGAGACCCCCTGGCAGCCAAGTAAGGCCGCCAGTGGGACAGAGGCACAGCCGCATCT  6571
             ||||||||||||||||||||||||||||||||||||||||||||||||||||||||||||
Sbjct  4801  CGGGGAGACCCCCTGGCAGCCAAGTAAGGCCGCCAGTGGGACAGAGGCACAGCCGCATCT  4860

Query  6572  GGACGTACTCAGAGCCAGTAATCGGAGGGCGGCCATGTTGGCGCGCTTTAAAGAAGGCTT  6631
             ||||||||||||||||||||||||||||||||||||||||||||||||||||||||||||
Sbjct  4861  GGACGTACTCAGAGCCAGTAATCGGAGGGCGGCCATGTTGGCGCGCTTTAAAGAAGGCTT  4920

Query  6632  CGGTGTCAGCTTTTCTGAGCTCACGCGCAGCTTTAAAAGCGACCGCTCCTGTGTGGGGGA  6691
             ||||||||||||||||||||||||||||||||||||||||||||||||||||||||||||
Sbjct  4921  CGGTGTCAGCTTTTCTGAGCTCACGCGCAGCTTTAAAAGCGACCGCTCCTGTGTGGGGGA  4980

Query  6692  CTGGGTCGTGCTGGCGTTTGGCGTCCGAGAACAGTTTGCCGAAACCGCCAAAGAGCAGCT  6751
             ||||||||||||||||||||||||||||||||||||||||||||||||||||||||||||
Sbjct  4981  CTGGGTCGTGCTGGCGTTTGGCGTCCGAGAACAGTTTGCCGAAACCGCCAAAGAGCAGCT  5040

Query  6752  GAAGGGACACTGTGGATACGTGCAGTTCACGTATCGACCGGACGCTAGGGGTGCGCTTAC  6811
             ||||||||||||||||||||||||||||||||||||||||||||||||||||||||||||
Sbjct  5041  GAAGGGACACTGTGGATACGTGCAGTTCACGTATCGACCGGACGCTAGGGGTGCGCTTAC  5100

Query  6812  ACTAGCGCTGCTGTCATTTACCTGTCAAAAGAATAGGGACACTGTACGTAACCTGATGAA  6871
             ||||||||||||||||||||||||||||||||||||||||||||||||||||||||||||
Sbjct  5101  ACTAGCGCTGCTGTCATTTACCTGTCAAAAGAATAGGGACACTGTACGTAACCTGATGAA  5160

Query  6872  AACTGTGTTAAATGTGCCAATAATCCAGATGCTCGCCGACCCTCCCCGCCTCCGTAGCAT  6931
             ||||||||||||||||||||||||||||||||||||||||||||||||||||||||||||
Sbjct  5161  AACTGTGTTAAATGTGCCAATAATCCAGATGCTCGCCGACCCTCCCCGCCTCCGTAGCAT  5220

Query  6932  GGCCTCCGCCCTGTTCTGGTTTAAGGGCAGCATGTCCAATTGTACATTTACACATGGGAT  6991
             ||||||||||||||||||||||||||||||||||||||||||||||||||||||||||||
Sbjct  5221  GGCCTCCGCCCTGTTCTGGTTTAAGGGCAGCATGTCCAATTGTACATTTACACATGGGAT  5280

Query  6992  TATGCCGCAGTGGCTGCGAGCACAAACCATGCTTAGCCAACAGACTGAGGAGGCGGTCAA  7051
             ||||||||||||||||||||||||||||||||||||||||||||||||||||||||||||
Sbjct  5281  TATGCCGCAGTGGCTGCGAGCACAAACCATGCTTAGCCAACAGACTGAGGAGGCGGTCAA  5340

Query  7052  ATTTGACCTGTCAGACATGATACAGTGGGCGCTGGACAATGACATAACTGAGGAGAGCAA  7111
             ||||||||||||||||||||||||||||||||||||||||||||||||||||||||||||
Sbjct  5341  ATTTGACCTGTCAGACATGATACAGTGGGCGCTGGACAATGACATAACTGAGGAGAGCAA  5400

Query  7112  ATTGGCATATGGGTACGCCCTCCTGGCCGAGAGCGACCCCAATGCAGCCGCCTTCCTGTC  7171
             ||||||||||||||||||||||||||||||||||||||||||||||||||||||||||||
Sbjct  5401  ATTGGCATATGGGTACGCCCTCCTGGCCGAGAGCGACCCCAATGCAGCCGCCTTCCTGTC  5460

Query  7172  CTCCAACAACCAGGCAAAACACGTGCGTGACGCGGCCACTATGGTCCGCCATTACAAGCG  7231
             ||||||||||||||||||||||||||||||||||||||||||||||||||||||||||||
Sbjct  5461  CTCCAACAACCAGGCAAAACACGTGCGTGACGCGGCCACTATGGTCCGCCATTACAAGCG  5520

Query  7232  GGCACAGATGCTGACCATGTCAATGTCCGCATGGGTGCACAGGCGGTGTGAGGCGGTGGA  7291
             ||||||||||||||||||||||||||||||||||||||||||||||||||||||||||||
Sbjct  5521  GGCACAGATGCTGACCATGTCAATGTCCGCATGGGTGCACAGGCGGTGTGAGGCGGTGGA  5580

Query  7292  GGAGCAAGGGGACTGGCGACCAATTATTTATTTTCTGAAATATCAGAATGTGGAAATTGC  7351
             ||||||||||||||||||||||||||||||||||||||||||||||||||||||||||||
```

```
Sbjct  5581  GGAGCAAGGGGACTGGCGACCAATTATTTATTTTCTGAAATATCAGAATGTGGAAATTGC  5640

Query  7352  TGCATTTTTGCAGTGCCTCAAACAGTTCTTTAAAGGGGTCCCCAAAAGAAATTGTCTAGT  7411
             ||||||||||||||||||||||||||||||||||||||||||||||||||||||||||||
Sbjct  5641  TGCATTTTTGCAGTGCCTCAAACAGTTCTTTAAAGGGGTCCCCAAAAGAAATTGTCTAGT  5700

Query  7412  AATCCAGGGACCACCAAACACAGGCAAGTCCACCTTCTGTATGAGCCTCATCAATTTCCT  7471
             ||||||||||||||||||||||||||||||||||||||||||||||||||||||||||||
Sbjct  5701  AATCCAGGGACCACCAAACACAGGCAAGTCCACCTTCTGTATGAGCCTCATCAATTTCCT  5760

Query  7472  TGCAGGGAAGGTGCTGTCCTTTGTTAATAGCAGGAGCCAGTTCTGGCTTATGCCCCTAGC  7531
             ||||||||||||||||||||||||||||||||||||||||||||||||||||||||||||
Sbjct  5761  TGCAGGGAAGGTGCTGTCCTTTGTTAATAGCAGGAGCCAGTTCTGGCTTATGCCCCTAGC  5820

Query  7532  TGAGACCAAGGT  7543
             ||||||||||||
Sbjct  5821  TGAGACCAAGGT  5832
```

Range 2: 5842 to 7607

Score:3262 bits(1766), Expect:0.0,
Identities:1766/1766(100%), Gaps:0/1766(0%), Strand: Plus/Plus

```
Query  1     GGACGATGCAACCATACCTGCCTGGGATTACCTAGACACATTCATGAGGAATGCCATGGA  60
             ||||||||||||||||||||||||||||||||||||||||||||||||||||||||||||
Sbjct  5842  GGACGATGCAACCATACCTGCCTGGGATTACCTAGACACATTCATGAGGAATGCCATGGA  5901

Query  61    CGGGAACCCCATCTGTATAGACCTGAAGCATCGGGCGCCCGTCCAAACACGCTGCCCCCC  120
             ||||||||||||||||||||||||||||||||||||||||||||||||||||||||||||
Sbjct  5902  CGGGAACCCCATCTGTATAGACCTGAAGCATCGGGCGCCCGTCCAAACACGCTGCCCCCC  5961

Query  121   TTTGTTAGTAACCACTAATGTAGACGTGGCAAACAACCCCAGATATCAATACCTCCACAC  180
             ||||||||||||||||||||||||||||||||||||||||||||||||||||||||||||
Sbjct  5962  TTTGTTAGTAACCACTAATGTAGACGTGGCAAACAACCCCAGATATCAATACCTCCACAC  6021

Query  181   ACGGATGGCCTGTTTCACCTTTCCTAATGACTTTATCTTAGATGAGAATGGGCAGCCTGT  240
             ||||||||||||||||||||||||||||||||||||||||||||||||||||||||||||
Sbjct  6022  ACGGATGGCCTGTTTCACCTTTCCTAATGACTTTATCTTAGATGAGAATGGGCAGCCTGT  6081

Query  241   GCACCAACTAACTGTAGCCAATTGGGCCTGTTTTTTCAAAAGGTTGTGGAGCAGATTAGA  300
             ||||||||||||||||||||||||||||||||||||||||||||||||||||||||||||
Sbjct  6082  GCACCAACTAACTGTAGCCAATTGGGCCTGTTTTTTCAAAAGGTTGTGGAGCAGATTAGA  6141

Query  301   GTTCAGTGAACAGGAAGACGAGGGCGAAGATGGAGAAGCTCCACAGACACTTAGAGTCTG  360
             ||||||||||||||||||||||||||||||||||||||||||||||||||||||||||||
Sbjct  6142  GTTCAGTGAACAGGAAGACGAGGGCGAAGATGGAGAAGCTCCACAGACACTTAGAGTCTG  6201

Query  361   TTACAGAAAGGCAGATGGAGATGTATGAAAGGGGCAGCACACGCCTGATTGATCAGGTGG  420
             ||||||||||||||||||||||||||||||||||||||||||||||||||||||||||||
Sbjct  6202  TTACAGAAAGGCAGATGGAGATGTATGAAAGGGGCAGCACACGCCTGATTGATCAGGTGG  6261

Query  421   AGCACTGGACGCTGCAAAGGAGAGAGGCTGTGCTTCTGCATGCTGCCAGGAAGCGGGGCC  480
             ||||||||||||||||||||||||||||||||||||||||||||||||||||||||||||
Sbjct  6262  AGCACTGGACGCTGCAAAGGAGAGAGGCTGTGCTTCTGCATGCTGCCAGGAAGCGGGGCC  6321

Query  481   TACACAGAGTTGGGCCATTTCCGGTGCCCAATGCGGCCACCTCAGCTGCCAATGCCAAAA  540
             ||||||||||||||||||||||||||||||||||||||||||||||||||||||||||||
Sbjct  6322  TACACAGAGTTGGGCCATTTCCGGTGCCCAATGCGGCCACCTCAGCTGCCAATGCCAAAA  6381

Query  541   CTGCCATCTTCATGCAGCTGACCCTTGAGTCGCTCCTCCACTCGCAGTTTGCAGAGGAGC  600
             ||||||||||||||||||||||||||||||||||||||||||||||||||||||||||||
```

```
Sbjct  6382  CTGCCATCTTCATGCAGCTGACCCTTGAGTCGCTCCTCCACTCGCAGTTTGCAGAGGAGC  6441

Query  601   CCTGGACCATGTCAGAGACCACAGCTGACATGTTTAACTGCCCGCCTAAGGACACGCTTA  660
             ||||||||||||||||||||||||||||||||||||||||||||||||||||||||||||
Sbjct  6442  CCTGGACCATGTCAGAGACCACAGCTGACATGTTTAACTGCCCGCCTAAGGACACGCTTA  6501

Query  661   AAAAGGACGGCTTTGTTGTGCAGGTGAAATTTGACAAGCAGGACGAAAACAGGATGTGGT  720
             ||||||||||||||||||||||||||||||||||||||||||||||||||||||||||||
Sbjct  6502  AAAAGGACGGCTTTGTTGTGCAGGTGAAATTTGACAAGCAGGACGAAAACAGGATGTGGT  6561

Query  721   ACACAGCATGGGGCAGCATTTACTACAGGAACCTTGAGGGGGACTGGGTGAAGACGCAGG  780
             ||||||||||||||||||||||||||||||||||||||||||||||||||||||||||||
Sbjct  6562  ACACAGCATGGGGCAGCATTTACTACAGGAACCTTGAGGGGGACTGGGTGAAGACGCAGG  6621

Query  781   GTAAGGCGAACGCCCTGGGCCTCTACTATGAGGCGGACGGGGaaaaaaaaTACTATGTAC  840
             ||||||||||||||||||||||||||||||||||||||||||||||||||||||||||||
Sbjct  6622  GTAAGGCGAACGCCCTGGGCCTCTACTATGAGGCGGACGGGGAAAAAAAATACTATGTAC  6681

Query  841   AGTTTGGTGATGAGGCCGCAAAGTATGGCACCTCCAGAAGCTGGGAGGTTTCCTATCGCA  900
             ||||||||||||||||||||||||||||||||||||||||||||||||||||||||||||
Sbjct  6682  AGTTTGGTGATGAGGCCGCAAAGTATGGCACCTCCAGAAGCTGGGAGGTTTCCTATCGCA  6741

Query  901   CTGAAACCTTCACCTATCCTTTCAACAGCGGATGCGGTGCAGCCGGACCCACAGAACCGA  960
             ||||||||||||||||||||||||||||||||||||||||||||||||||||||||||||
Sbjct  6742  CTGAAACCTTCACCTATCCTTTCAACAGCGGATGCGGTGCAGCCGGACCCACAGAACCGA  6801

Query  961   TTCCAGGAGGACAGCCCACACCTCCAGCTACCCCTGACTCGCCGGGACTTCCTTTTGCAG  1020
             ||||||||||||||||||||||||||||||||||||||||||||||||||||||||||||
Sbjct  6802  TTCCAGGAGGACAGCCCACACCTCCAGCTACCCCTGACTCGCCGGGACTTCCTTTTGCAG  6861

Query  1021  CAACAACTTTTGGGCGGAGACATTTGGCCACATCAGACGGGGGCGCAAGTGAACCAAAGA  1080
             ||||||||||||||||||||||||||||||||||||||||||||||||||||||||||||
Sbjct  6862  CAACAACTTTTGGGCGGAGACATTTGGCCACATCAGACGGGGGCGCAAGTGAACCAAAGA  6921

Query  1081  GGTACTGCCCTCATCCTGACACTGTCAGCTGCGACAGCCACAGAGGGGGAGAGGGCCAGT  1140
             ||||||||||||||||||||||||||||||||||||||||||||||||||||||||||||
Sbjct  6922  GGTACTGCCCTCATCCTGACACTGTCAGCTGCGACAGCCACAGAGGGGGAGAGGGCCAGT  6981

Query  1141  GTACCCTCTCCGGGACCACGGGACGAAGCCCAGGGCTACTATTGCATCCAccccccgccc  1200
             ||||||||||||||||||||||||||||||||||||||||||||||||||||||||||||
Sbjct  6982  GTACCCTCTCCGGGACCACGGGACGAAGCCCAGGGCTACTATTGCATCCACCCCCCGCCC  7041

Query  1201  cccGCCTGCAGGCGGCGGATGAGGCGGACCCGGCACAGACAGAGGCAGCAGCTCCGTCGG  1260
             ||||||||||||||||||||||||||||||||||||||||||||||||||||||||||||
Sbjct  7042  CCCGCCTGCAGGCGGCGGATGAGGCGGACCCGGCACAGACAGAGGCAGCAGCTCCGTCGG  7101

Query  1261  ACCCTGGCAGACCTAAGGCAACAGCTTCAGGAGCAGGGGCTTCTCCTTCGCATCGAGATA  1320
             ||||||||||||||||||||||||||||||||||||||||||||||||||||||||||||
Sbjct  7102  ACCCTGGCAGACCTAAGGCAACAGCTTCAGGAGCAGGGGCTTCTCCTTCGCATCGAGATA  7161

Query  1321  CTGTAGGACCTCTCTTCCTATGCTTGTCAGGGGGCGCCAATAGCCTGAAGTGCCTGCGTT  1380
             ||||||||||||||||||||||||||||||||||||||||||||||||||||||||||||
Sbjct  7162  CTGTAGGACCTCTCTTCCTATGCTTGTCAGGGGGCGCCAATAGCCTGAAGTGCCTGCGTT  7221

Query  1381  TCCGGTGCAAAAAGTACAAAGCCTCCTTGTTTGACTCTATGTCCACAACATTCTTTTGGA  1440
             ||||||||||||||||||||||||||||||||||||||||||||||||||||||||||||
Sbjct  7222  TCCGGTGCAAAAAGTACAAAGCCTCCTTGTTTGACTCTATGTCCACAACATTCTTTTGGA  7281

Query  1441  CGGGACCTGAGGGCAAGGAGAGAGTGGGTGAAGCCCGTGTGCTGGTTACATTCTCGGGGG  1500
             ||||||||||||||||||||||||||||||||||||||||||||||||||||||||||||
Sbjct  7282  CGGGACCTGAGGGCAAGGAGAGAGTGGGTGAAGCCCGTGTGCTGGTTACATTCTCGGGGG  7341

Query  1501  CACAGCAGAAGCAGGACTTCATGGAGAAGGTCTCACTGCCCCCCGGTGTGCGGGCGAGCC  1560
             ||||||||||||||||||||||||||||||||||||||||||||||||||||||||||||
```

```
Sbjct  7342  CACAGCAGAAGCAGGACTTCATGGAGAAGGTCTCACTGCCCCCCGGTGTGCGGGCGAGCC  7401

Query  1561  AGGCTGTCATGTGACTGTGGACATGGTACTGGACCTTGTGCATGCTGCTGTTTTGATTTT  1620
             ||||||||||||||||||||||||||||||||||||||||||||||||||||||||||||
Sbjct  7402  AGGCTGTCATGTGACTGTGGACATGGTACTGGACCTTGTGCATGCTGCTGTTTTGATTTT  7461

Query  1621  GTGCCTTGTTGTGTTGTTGGAGTATTGCTTGGtttttttACTTGTTATTTTAGTAAATTT  1680
             ||||||||||||||||||||||||||||||||||||||||||||||||||||||||||||
Sbjct  7462  GTGCCTTGTTGTGTTGTTGGAGTATTGCTTGGTTTTTTTACTTGTTATTTTAGTAAATTT  7521

Query  1681  TCACTAAAACCTGTGTCATGGCGCTGCTTGccccccccccAGGGAAGCGGCGGCGCCGCG  1740
             ||||||||||||||||||||||||||||||||||||||||||||||||||||||||||||
Sbjct  7522  TCACTAAAACCTGTGTCATGGCGCTGCTTGCCCCCCCCCCAGGGAAGCGGCGGCGCCGCG  7581

Query  1741  CCGCGGAGGGTGACCTGTACCGAGGG  1766
             ||||||||||||||||||||||||||
Sbjct  7582  CCGCGGAGGGTGACCTGTACCGAGGG  7607
```

##
##
RID: NR7D5NFW114
Job Title:NODE_183_length_7543_cov_4.284892
Program: BLASTN
Query: NODE_183_length_7543_cov_4.284892 ID: lcl|Query_51579(dna) Length: 7543
Subject:NODE_328_length_5539_cov_3.362871 ID: lcl|Query_51581(dna) Length: 5539

Sequences producing significant alignments:

```
                                                                         Scientific
Common                    Max    Total Query   E   Per.   Acc.
Description                                                              Name
Name           Taxid      Score  Score cover Value Ident  Len       Accession
NODE_328_length_5539_cov_3.362871
0          9084   10197 73%   0.0   99.94  5539       Query_51581
```

Alignments:

>NODE_328_length_5539_cov_3.362871
Sequence ID: Query_51581 Length: 5539
Range 1: 612 to 5539

Score:9084 bits(4919), Expect:0.0,
Identities:4925/4928(99%), Gaps:0/4928(0%), Strand: Plus/Plus

```
Query  1     GGACGATGCAACCATACCTGCCTGGGATTACCTAGACACATTCATGAGGAATGCCATGGA  60
             ||||||||||||||||||||||||||||||||||||||||||||||||||||||||||||
Sbjct  612   GGACGATGCAACCATACCTGCCTGGGATTACCTAGACACATTCATGAGGAATGCCATGGA  671

Query  61    CGGGAACCCCATCTGTATAGACCTGAAGCATCGGGCGCCCGTCCAAACACGCTGCCCCCC  120
             ||||||||||||||||||||||||||||||||||||||||||||||||||||||||||||
Sbjct  672   CGGGAACCCCATCTGTATAGACCTGAAGCATCGGGCGCCCGTCCAAACACGCTGCCCCCC  731

Query  121   TTTGTTAGTAACCACTAATGTAGACGTGGCAAACAACCCCAGATATCAATACCTCCACAC  180
             ||||||||||||||||||||||||||||||||||||||||||||||||||||||||||||
Sbjct  732   TTTGTTAGTAACCACTAATGTAGACGTGGCAAACAACCCCAGATATCAATACCTCCACAC  791

Query  181   ACGGATGGCCTGTTTCACCTTTCCTAATGACTTTATCTTAGATGAGAATGGGCAGCCTGT  240
             ||||||||||||||||||||||||||||||||||||||||||||||||||||||||||||
```

```
Sbjct  792   ACGGATGGCCTGTTTCACCTTTCCTAATGACTTTATCTTAGATGAGAATGGGCAGCCTGT  851

Query  241   GCACCAACTAACTGTAGCCAATTGGGCCTGTTTTTTCAAAAGGTTGTGGAGCAGATTAGA  300
             ||||||||||||||||||||||||||||||||||||||||||||||||||||||||||||
Sbjct  852   GCACCAACTAACTGTAGCCAATTGGGCCTGTTTTTTCAAAAGGTTGTGGAGCAGATTAGA  911

Query  301   GTTCAGTGAACAGGAAGACGAGGGCGAAGATGGAGAAGCTCCACAGACACTTAGAGTCTG  360
             ||||||||||||||||||||||||||||||||||||||||||||||||||||||||||||
Sbjct  912   GTTCAGTGAACAGGAAGACGAGGGCGAAGATGGAGAAGCTCCACAGACACTTAGAGTCTG  971

Query  361   TTACAGAAAGGCAGATGGAGATGTATGAAAGGGGCAGCACACGCCTGATTGATCAGGTGG  420
             ||||||||||||||||||||||||||||||||||||||||||||||||||||||||||||
Sbjct  972   TTACAGAAAGGCAGATGGAGATGTATGAAAGGGGCAGCACACGCCTGATTGATCAGGTGG  1031

Query  421   AGCACTGGACGCTGCAAAGGAGAGAGGCTGTGCTTCTGCATGCTGCCAGGAAGCGGGGCC  480
             ||||||||||||||||||||||||||||||||||||||||||||||||||||||||||||
Sbjct  1032  AGCACTGGACGCTGCAAAGGAGAGAGGCTGTGCTTCTGCATGCTGCCAGGAAGCGGGGCC  1091

Query  481   TACACAGAGTTGGGCCATTTCCGGTGCCCAATGCGGCCACCTCAGCTGCCAATGCCAAAA  540
             ||||||||||||||||||||||||||||||||||||||||||||||||||||||||||||
Sbjct  1092  TACACAGAGTTGGGCCATTTCCGGTGCCCAATGCGGCCACCTCAGCTGCCAATGCCAAAA  1151

Query  541   CTGCCATCTTCATGCAGCTGACCCTTGAGTCGCTCCTCCACTCGCAGTTTGCAGAGGAGC  600
             ||||||||||||||||||||||||||||||||||||||||||||||||||||||||||||
Sbjct  1152  CTGCCATCTTCATGCAGCTGACCCTTGAGTCGCTCCTCCACTCGCAGTTTGCAGAGGAGC  1211

Query  601   CCTGGACCATGTCAGAGACCACAGCTGACATGTTTAACTGCCCGCCTAAGGACACGCTTA  660
             ||||||||||||||||||||||||||||||||||||||||||||||||||||||||||||
Sbjct  1212  CCTGGACCATGTCAGAGACCACAGCTGACATGTTTAACTGCCCGCCTAAGGACACGCTTA  1271

Query  661   AAAAGGACGGCTTTGTTGTGCAGGTGAAATTTGACAAGCAGGACGAAAACAGGATGTGGT  720
             ||||||||||||||||||||||||||||||||||||||||||||||||||||||||||||
Sbjct  1272  AAAAGGACGGCTTTGTTGTGCAGGTGAAATTTGACAAGCAGGACGAAAACAGGATGTGGT  1331

Query  721   ACACAGCATGGGGCAGCATTTACTACAGGAACCTTGAGGGGGACTGGGTGAAGACGCAGG  780
             ||||||||||||||||||||||||||||||||||||||||||||||||||||||||||||
Sbjct  1332  ACACAGCATGGGGCAGCATTTACTACAGGAACCTTGAGGGGGACTGGGTGAAGACGCAGG  1391

Query  781   GTAAGGCGAACGCCCTGGGCCTCTACTATGAGGCGGACGGGGaaaaaaaaTACTATGTAC  840
             ||||||||||||||||||||||||||||||||||||||||||||||||||||||||||||
Sbjct  1392  GTAAGGCGAACGCCCTGGGCCTCTACTATGAGGCGGACGGGGAAAAAAAATACTATGTAC  1451

Query  841   AGTTTGGTGATGAGGCCGCAAAGTATGGCACCTCCAGAAGCTGGGAGGTTTCCTATCGCA  900
             ||||||||||||||||||||||||||||||||||||||||||||||||||||||||||||
Sbjct  1452  AGTTTGGTGATGAGGCCGCAAAGTATGGCACCTCCAGAAGCTGGGAGGTTTCCTATCGCA  1511

Query  901   CTGAAACCTTCACCTATCCTTTCAACAGCGGATGCGGTGCAGCCGGACCCACAGAACCGA  960
             ||||||||||||||||||||||||||||||||||||||||||||||||||||||||||||
Sbjct  1512  CTGAAACCTTCACCTATCCTTTCAACAGCGGATGCGGTGCAGCCGGACCCACAGAACCGA  1571

Query  961   TTCCAGGAGGACAGCCCACACCTCCAGCTACCCCTGACTCGCCGGGACTTCCTTTTGCAG  1020
             ||||||||||||||||||||||||||||||||||||||||||||||||||||||||||||
Sbjct  1572  TTCCAGGAGGACAGCCCACACCTCCAGCTACCCCTGACTCGCCGGGACTTCCTTTTGCAG  1631

Query  1021  CAACAACTTTTGGGCGGAGACATTTGGCCACATCAGACGGGGGCGCAAGTGAACCAAAGA  1080
             ||||||||||||||||||||||||||||||||||||||||||||||||||||||||||||
Sbjct  1632  CAACAACTTTTGGGCGGAGACATTTGGCCACATCAGACGGGGGCGCAAGTGAACCAAAGA  1691

Query  1081  GGTACTGCCCTCATCCTGACACTGTCAGCTGCGACAGCCACAGAGGGGGAGAGGGCCAGT  1140
             ||||||||||||||||||||||||||||||||||||||||||||||||||||||||||||
Sbjct  1692  GGTACTGCCCTCATCCTGACACTGTCAGCTGCGACAGCCACAGAGGGGGAGAGGGCCAGT  1751

Query  1141  GTACCCTCTCCGGGACCACGGGACGAAGCCCAGGGCTACTATTGCATCCAcccccgccc  1200
             ||||||||||||||||||||||||||||||||||||||||||||||||||||||||||||
```

```
Sbjct  1752  GTACCCTCTCCGGGACCACGGGACGAAGCCCAGGGCTACTATTGCATCCACCCCCCGCCC  1811

Query  1201  cccGCCTGCAGGCGGCGGATGAGGCGGACCCGGCACAGACAGAGGCAGCAGCTCCGTCGG  1260
             ||||||||||||||||||||||||||||||||||||||||||||||||||||||||||||
Sbjct  1812  CCCGCCTGCAGGCGGCGGATGAGGCGGACCCGGCACAGACAGAGGCAGCAGCTCCGTCGG  1871

Query  1261  ACCCTGGCAGACCTAAGGCAACAGCTTCAGGAGCAGGGGCTTCTCCTTCGCATCGAGATA  1320
             ||||||||||||||||||||||||||||||||||||||||||||||||||||||||||||
Sbjct  1872  ACCCTGGCAGACCTAAGGCAACAGCTTCAGGAGCAGGGGCTTCTCCTTCGCATCGAGATA  1931

Query  1321  CTGTAGGACCTCTCTTCCTATGCTTGTCAGGGGGCGCCAATAGCCTGAAGTGCCTGCGTT  1380
             ||||||||||||||||||||||||||||||||||||||||||||||||||||||||||||
Sbjct  1932  CTGTAGGACCTCTCTTCCTATGCTTGTCAGGGGGCGCCAATAGCCTGAAGTGCCTGCGTT  1991

Query  1381  TCCGGTGCAAAAAGTACAAAGCCTCCTTGTTTGACTCTATGTCCACAACATTCTTTTGGA  1440
             ||||||||||||||||||||||||||||||||||||||||||||||||||||||||||||
Sbjct  1992  TCCGGTGCAAAAAGTACAAAGCCTCCTTGTTTGACTCTATGTCCACAACATTCTTTTGGA  2051

Query  1441  CGGGACCTGAGGGCAAGGAGAGAGTGGGTGAAGCCCGTGTGCTGGTTACATTCTCGGGGG  1500
             ||||||||||||||||||||||||||||||||||||||||||||||||||||||||||||
Sbjct  2052  CGGGACCTGAGGGCAAGGAGAGAGTGGGTGAAGCCCGTGTGCTGGTTACATTCTCGGGGG  2111

Query  1501  CACAGCAGAAGCAGGACTTCATGGAGAAGGTCTCACTGCCCCCCGGTGTGCGGGCGAGCC  1560
             ||||||||||||||||||||||||||||||||||||||||||||||||||||||||||||
Sbjct  2112  CACAGCAGAAGCAGGACTTCATGGAGAAGGTCTCACTGCCCCCCGGTGTGCGGGCGAGCC  2171

Query  1561  AGGCTGTCATGTGACTGTGGACATGGTACTGGACCTTGTGCATGCTGCTGTTTTGATTTT  1620
             ||||||||||||||||||||||||||||||||||||||||||||||||||||||||||||
Sbjct  2172  AGGCTGTCATGTGACTGTGGACATGGTACTGGACCTTGTGCATGCTGCTGTTTTGATTTT  2231

Query  1621  GTGCCTTGTTGTGTTGTTGGAGTATTGCTTGGtttttttACTTGTTATTTTAGTAAATTT  1680
             ||||||||||||||||||||||||||||||||||||||||||||||||||||||||||||
Sbjct  2232  GTGCCTTGTTGTGTTGTTGGAGTATTGCTTGGTTTTTTTACTTGTTATTTTAGTAAATTT  2291

Query  1681  TCACTAAAACCTGTGTCATGGCGCTGCTTGcccccccccccAGGGAAGCGGCGGCGCCGCG  1740
             ||||||||||||||||||||||||||||||||||||||||||||||||||||||||||||
Sbjct  2292  TCACTAAAACCTGTGTCATGGCGCTGCTTGCCCCCCCCCCCAGGGAAGCGGCGGCGCCGCG  2351

Query  1741  CCGCGGAGGGTGACCTGTACCGAGGGTGCATTATGGGGCAGGACTGCCCGCCGGACATCA  1800
             ||||||||||||||||||||||||||||||||||||||||||||||||||||||||||||
Sbjct  2352  CCGCGGAGGGTGACCTGTACCGAGGGTGCATTATGGGGCAGGACTGCCCGCCGGACATCA  2411

Query  1801  AGGCAAAGTTTGAGAACGACACACTTGCAGACCGCTTTCTAAAGTGGGTCAGCTCCTTCC  1860
             ||||||||||||||||||||||||||||||||||||||||||||||||||||||||||||
Sbjct  2412  AGGCAAAGTTTGAGAACGACACACTTGCAGACCGCTTTCTAAAGTGGGTCAGCTCCTTCC  2471

Query  1861  TCTACCTAGGCAACCTGGGTTTCAGCACGGGCAGGGGCAATCCCAGGTTTGGGTACAGAC  1920
             ||||||||||||||||||||||||||||||||||||||||||||||||||||||||||||
Sbjct  2472  TCTACCTAGGCAACCTGGGTTTCAGCACGGGCAGGGGCAATCCCAGGTTTGGGTACAGAC  2531

Query  1921  CTGTgggggcggggtaggcgaggcgggtgggggggtgcgggtcgggACGCCCATTACGG  1980
             |||||||||||||||||||||||||||| ||||||| |||||||||||||||||||||||
Sbjct  2532  CTGTGGGGGCGGGGTAGGCGAGGCGGGGGGGGGGGGGCGGGTCGGGACGCCCATTACGG  2591

Query  1981  ACGTGTCCCCGGGGGTCACTGTGGAGACGGTGGGGCCCGCGGAGGTGGTCCCAGTAGACA  2040
             ||||||||||||||||||||||||||||||||||||||||||||||||||||||||||||
Sbjct  2592  ACGTGTCCCCGGGGGTCACTGTGGAGACGGTGGGGCCCGCGGAGGTGGTCCCAGTAGACA  2651

Query  2041  GTTTAAGCCCCTCAGCCCCGGCTGTGGTGCCTGCGGGGGAAGGCACAGGGGGCGCTGTGG  2100
             ||||||||||||||||||||||||||||||||||||||||||||||||||||||||||||
Sbjct  2652  GTTTAAGCCCCTCAGCCCCGGCTGTGGTGCCTGCGGGGGAAGGCACAGGGGGCGCTGTGG  2711

Query  2101  ATGTGGAGTTGGTGGCTGAGGTGCACCCTACCGTGGACCCGGGCATGGCTGGGGGCTCGG  2160
             ||||||||||||||||||||||||||||||||||||||||||||||||||||||||||||
```

```
Sbjct  2712  ATGTGGAGTTGGTGGCTGAGGTGCACCCTACCGTGGACCCGGGCATGGCTGGGGGCTCGG  2771

Query  2161  GGGGTGTTACCAGCAACGGCGGGGATGCCGCCGTGCTGGAGGTGCCCCCTGAGGTGGTTC  2220
             ||||||||||||||||||||||||||||||||||||||||||||||||||||||||||||
Sbjct  2772  GGGGTGTTACCAGCAACGGCGGGGATGCCGCCGTGCTGGAGGTGCCCCCTGAGGTGGTTC  2831

Query  2221  CCCGGGGTCGGAATGCTACTAGCCGCACACAGTTTCATAATCCTGCATTCCATGTGGAGC  2280
             ||||||||||||||||||||||||||||||||||||||||||||||||||||||||||||
Sbjct  2832  CCCGGGGTCGGAATGCTACTAGCCGCACACAGTTTCATAATCCTGCATTCCATGTGGAGC  2891

Query  2281  TAAACAGCTCGCTCCCCACGGGTGAATCGTCCGCCTCTGACCATGTGTTTGTCCAGGCGG  2340
             ||||||||||||||||||||||||||||||||||||||||||||||||||||||||||||
Sbjct  2892  TAAACAGCTCGCTCCCCACGGGTGAATCGTCCGCCTCTGACCATGTGTTTGTCCAGGCGG  2951

Query  2341  AACAGGGTGGTCACTGGGTGGGAGAGGAAATTGAATTGCTTCCTTTGGGCGATACGCTGA  2400
             ||||||||||||||||||||||||||||||||||||||||||||||||||||||||||||
Sbjct  2952  AACAGGGTGGTCACTGGGTGGGAGAGGAAATTGAATTGCTTCCTTTGGGCGATACGCTGA  3011

Query  2401  CGCAAAGAACCAGCACCCCCAGAACTGGGGCACGGGGACGCGCAGAGAGCGGGGCGGAGC  2460
             ||||||||||||||||||||||||||||||||||||||||||||||||||||||||||||
Sbjct  3012  CGCAAAGAACCAGCACCCCCAGAACTGGGGCACGGGGACGCGCAGAGAGCGGGGCGGAGC  3071

Query  2461  GTCTATTTGGACGTGGCTTCCGTCAGGTGCGCGTAACCGATCCAAACTTCCTCTCCGACC  2520
             ||||||||||||||||||||||||||||||||||||||||||||||||||||||||||||
Sbjct  3072  GTCTATTTGGACGTGGCTTCCGTCAGGTGCGCGTAACCGATCCAAACTTCCTCTCCGACC  3131

Query  2521  CTGGGTCTCTTGTGCAGTTTGGGTTCGAGAACCCTGCATATGACCCCTCCGGCAGCATCT  2580
             ||||||||||||||||||||||||||||||||||||||||||||||||||||||||||||
Sbjct  3132  CTGGGTCTCTTGTGCAGTTTGGGTTCGAGAACCCTGCATATGACCCCTCCGGCAGCATCT  3191

Query  2581  CCTTTGGGCCACTCGGCGAGCCGGAGGCGGCCCCCAACCCAGAGTTTCAGGATGTTGTGC  2640
             ||||||||||||||||||||||||||||||||||||||||||||||||||||||||||||
Sbjct  3192  CCTTTGGGCCACTCGGCGAGCCGGAGGCGGCCCCCAACCCAGAGTTTCAGGATGTTGTGC  3251

Query  2641  ACCTTGGCAGGACACACATTACAGAGCGCGACGGGCGCGTGGTGCTAGGAAGATTTGGTC  2700
             ||||||||||||||||||||||||||||||||||||||||||||||||||||||||||||
Sbjct  3252  ACCTTGGCAGGACACACATTACAGAGCGCGACGGGCGCGTGGTGCTAGGAAGATTTGGTC  3311

Query  2701  AGCGGGCTGGGGTGTCCACCCGCAGCGGGCGGGTGGTGGGACCCCAGGTACACTATAGGT  2760
             ||||||||||||||||||||||||||||||||||||||||||||||||||||||||||||
Sbjct  3312  AGCGGGCTGGGGTGTCCACCCGCAGCGGGCGGGTGGTGGGACCCCAGGTACACTATAGGT  3371

Query  2761  TTGAGTTCAGCAGCATTGCCCCTGCAGAGGAGATTGAACTGGTGCCTCTGCCAAGTGCGG  2820
             ||||||||||||||||||||||||||||||||||||||||||||||||||||||||||||
Sbjct  3372  TTGAGTTCAGCAGCATTGCCCCTGCAGAGGAGATTGAACTGGTGCCTCTGCCAAGTGCGG  3431

Query  2821  ATCCGCCGGGGTCACTTGGGGACGAGTTTGAGGTTATCGACCTGGACAGCACAGGTAGTG  2880
             ||||||||||||||||||||||||||||||||||||||||||||||||||||||||||||
Sbjct  3432  ATCCGCCGGGGTCACTTGGGGACGAGTTTGAGGTTATCGACCTGGACAGCACAGGTAGTG  3491

Query  2881  TGTACAGCGAGGCGGCTCTGCTGGACGATGACAGCCTCAGCATTCACGGGGTGCTTAGCT  2940
             ||||||||||||||||||||||||||||||||||||||||||||||||||||||||||||
Sbjct  3492  TGTACAGCGAGGCGGCTCTGCTGGACGATGACAGCCTCAGCATTCACGGGGTGCTTAGCT  3551

Query  2941  TGGGTCAGAGAGCTGGCAGCCACCGCGCTGTGTCCGTCCTGGACTTTGCTGGACCTAGAG  3000
             ||||||||||||||||||||||||||||||||||||||||||||||||||||||||||||
Sbjct  3552  TGGGTCAGAGAGCTGGCAGCCACCGCGCTGTGTCCGTCCTGGACTTTGCTGGACCTAGAG  3611

Query  3001  CCTTTTACAGTGCACTGACAGTAGAGGGCGACGTGGCACCTGCACACCCGAACCCCACTA  3060
             ||||||||||||||||||||||||||||||||||||||||||||||||||||||||||||
Sbjct  3612  CCTTTTACAGTGCACTGACAGTAGAGGGCGACGTGGCACCTGCACACCCGAACCCCACTA  3671

Query  3061  GCTCTATCCTTGTGGTCCCTGAGAACGCCGAGCAGACCCCCTCTGTGGTTGTTGAATCGA  3120
             ||||||||||||||||||||||||||||||||||||||||||||||||||||||||||||
```

```
Sbjct  3672  GCTCTATCCTTGTGGTCCCTGAGAACGCCGAGCAGACCCCCTCTGTGGTTGTTGAATCGA  3731

Query  3121  TGCCGGTCAGTGGCCTGTATGACCTGCACCCCAGCTTGCACCCTAGACGCAGGCGCAAAC  3180
             ||||||||||||||||||||||||||||||||||||||||||||||||||||||||||||
Sbjct  3732  TGCCGGTCAGTGGCCTGTATGACCTGCACCCCAGCTTGCACCCTAGACGCAGGCGCAAAC  3791

Query  3181  GCAGGCGTTCCTTCTGTTTTGCAGATGGCAGTGTGGACACAAAACAAGCAGAAGCTTTTT  3240
             ||||||||||||||||||||||||||||||||||||||||||||||||||||||||||||
Sbjct  3792  GCAGGCGTTCCTTCTGTTTTGCAGATGGCAGTGTGGACACAAAACAAGCAGAAGCTTTTT  3851

Query  3241  CTGCCTCCGCCGCCTGTTACTAGAGTCCCTAACACAGATGAGTATGTCGTGCGACACAAC  3300
             ||||||||||||||||||||||||||||||||||||||||||||||||||||||||||||
Sbjct  3852  CTGCCTCCGCCGCCTGTTACTAGAGTCCCTAACACAGATGAGTATGTCGTGCGACACAAC  3911

Query  3301  ATATTTTACCATGGTAACAGTGACCGACTGCTTATGGTCGGCCACCCTTTCTTTCCTGTG  3360
             ||||||||||||||||||||||||||||||||||||||||||||||||||||||||||||
Sbjct  3912  ATATTTTACCATGGTAACAGTGACCGACTGCTTATGGTCGGCCACCCTTTCTTTCCTGTG  3971

Query  3361  AAGAGTGGCAACATAGATGTTCCTAAGGTGTCTGCAAATCAATTCAGGGTCTTTAAGGTG  3420
             ||||||||||||||||||||||||||||||||||||||||||||||||||||||||||||
Sbjct  3972  AAGAGTGGCAACATAGATGTTCCTAAGGTGTCTGCAAATCAATTCAGGGTCTTTAAGGTG  4031

Query  3421  CTTCTGCCTGACCCTAATAAGTTTGCTCTGGCCGACCCTGACTTATACAACCCGGAGACG  3480
             ||||||||||||||||||||||||||||||||||||||||||||||||||||||||||||
Sbjct  4032  CTTCTGCCTGACCCTAATAAGTTTGCTCTGGCCGACCCTGACTTATACAACCCGGAGACG  4091

Query  3481  GGCCGCCTGGTATGGGCCCTAAAGGGCTTGGAGGTTAGCAGGGGCCAGCCCCTGGGCATA  3540
             |||||||||||||||||||||| |||||||||||||||||||||||||||||||||||||
Sbjct  4092  GGCCGCCTGGTATGGGCCCTAACGGGCTTGGAGGTTAGCAGGGGCCAGCCCCTGGGCATA  4151

Query  3541  GGTGTCACAGGTAACCCCCTGTTTAACAAAAAGAATGATGTTGAAAACCCTAGCAAGCTA  3600
             ||||||||||||||||||||||||||||||||||||||||||||||||||||||||||||
Sbjct  4152  GGTGTCACAGGTAACCCCCTGTTTAACAAAAAGAATGATGTTGAAAACCCTAGCAAGCTA  4211

Query  3601  CAGGGGGCAGGTGCCAAGGATGACAGGGTGAACATGGGCTTTGATGTGAAGCAACACCAG  3660
             ||||||||||||||||||||||||||||||||||||||||||||||||||||||||||||
Sbjct  4212  CAGGGGGCAGGTGCCAAGGATGACAGGGTGAACATGGGCTTTGATGTGAAGCAACACCAG  4271

Query  3661  CTGCTGCTGGTGGGTTGCAAGCCCCCACAGGGGGAGCACTGGAGGAAGACACGCTTTTGC  3720
             ||||||||||||||||||||||||||||||||||||||||||||||||||||||||||||
Sbjct  4272  CTGCTGCTGGTGGGTTGCAAGCCCCCACAGGGGGAGCACTGGAGGAAGACACGCTTTTGC  4331

Query  3721  AACACAGGACAGCCACCGGTGGGCTCCTGCCCGGCCATAGAGCTGGTCAATACCGTGATA  3780
             ||||||||||||||||||||||||||||||||||||||||||||||||||||||||||||
Sbjct  4332  AACACAGGACAGCCACCGGTGGGCTCCTGCCCGGCCATAGAGCTGGTCAATACCGTGATA  4391

Query  3781  GAGGATGGGGACATGGGGGACGTGGGTTTCGGGGCAATGGACTTTGCCACACTTTGTGAC  3840
             ||||||||||||||||||||||||||||||||||||||||||||||||||||||||||||
Sbjct  4392  GAGGATGGGGACATGGGGGACGTGGGTTTCGGGGCAATGGACTTTGCCACACTTTGTGAC  4451

Query  3841  AGTAAGGCCGATGTGCCTCTGGACCTCGTGGGCACTGCCTCGAAGTACCCCGACTACATT  3900
             ||||||||||||||||||||||||||||||||||||||||||||||||||||||||||||
Sbjct  4452  AGTAAGGCCGATGTGCCTCTGGACCTCGTGGGCACTGCCTCGAAGTACCCCGACTACATT  4511

Query  3901  AAGATGGGGCAGGAGCCTGCAGGGGACAGCATGTGGTTTTTCGCCAGGAGGGAGCAGTAC  3960
             ||||||||||||||||||||||||||||||||||||||||||||||||||||||||||||
Sbjct  4512  AAGATGGGGCAGGAGCCTGCAGGGGACAGCATGTGGTTTTTCGCCAGGAGGGAGCAGTAC  4571

Query  3961  TATGCCAGGCACTTTTTTACGCGGGACGGTAAGAGTCTGGAGACGGTGCCCCCCGAGTTG  4020
             ||||||||||||||||||||||||||||||||||||||||||||||||||||||||||||
Sbjct  4572  TATGCCAGGCACTTTTTTACGCGGGACGGTAAGAGTCTGGAGACGGTGCCCCCCGAGTTG  4631

Query  4021  TACACAGCACCAGAGGCAAACCCAACCAACATCAACAGGTACATTTACAGTGCAAGCCCC  4080
             ||||||||||||||||||||||||||||||||||||||||||||||||||||||||||||
```

```
Sbjct  4632  TACACAGCACCAGAGGCAAACCCAACCAACATCAACAGGTACATTTACAGTGCAAGCCCC  4691

Query  4081  AGTGGGTCTCTCGTGTCCACAGACTCACAGATATTTAACAGGCCGTACTGGCTGGCACGC  4140
             ||||||||||||||||||||||||||||||||||||||||||||||||||||||||||||
Sbjct  4692  AGTGGGTCTCTCGTGTCCACAGACTCACAGATATTTAACAGGCCGTACTGGCTGGCACGC  4751

Query  4141  GCACAGGGCCAGAATAATGGCATCTGCTGGCACAACACTTTGTATGTGACCGTGTTTGAC  4200
             ||||||||||||||||||||||||||||||||||||||||||||||||||||||||||||
Sbjct  4752  GCACAGGGCCAGAATAATGGCATCTGCTGGCACAACACTTTGTATGTGACCGTGTTTGAC  4811

Query  4201  AACACACGTGGCACAAACTTGACCATTACAGTGAACCCCCAGAACACATATGATTCCACA  4260
             ||||||||||||||||||||||||||||||||||||||||||||||||||||||||||||
Sbjct  4812  AACACACGTGGCACAAACTTGACCATTACAGTGAACCCCCAGAACACATATGATTCCACA  4871

Query  4261  GGCTTTAATGCATACGTGCGACATGTGGAGGAGTTTGAGCTCTCCTTTATCTTTCAGCTG  4320
             ||||||||||||||||||||||||||||||||||||||||||||||||||||||||||||
Sbjct  4872  GGCTTTAATGCATACGTGCGACATGTGGAGGAGTTTGAGCTCTCCTTTATCTTTCAGCTG  4931

Query  4321  TGCACGGTCCCCTTGACACCCCAGGTCCTTGCCCACCTCCATACAACCGATGCCAGCCTC  4380
             ||||||||||||||||||||||||||||||||||||||||||||||||||||||||||||
Sbjct  4932  TGCACGGTCCCCTTGACACCCCAGGTCCTTGCCCACCTCCATACAACCGATGCCAGCCTC  4991

Query  4381  CTTGAGGAGTGGGAGATTGGGGTAAAcccccccTCCTCCTCGCAGCTGGAGGACACATAC  4440
             ||||||||||||||||||||||||||||||||||||||||||||||||||||||||||||
Sbjct  4992  CTTGAGGAGTGGGAGATTGGGGTAAACCCCCCCTCCTCCTCGCAGCTGGAGGACACATAC  5051

Query  4441  CGGTTTATTACCTCTACTGCCACAAAGTGCCCTGTGCCTCCCCAGGCCCCGCTTGAGCCA  4500
             ||||||||||||||||||||||||||||||||||||||||||||||||||||||||||||
Sbjct  5052  CGGTTTATTACCTCTACTGCCACAAAGTGCCCTGTGCCTCCCCAGGCCCCGCTTGAGCCA  5111

Query  4501  CCGGGGTACACATTTTGGACTGTGGATCTGCAGGAGCGTTTGTCCTTGGACCTGGACCAG  4560
             ||||||||||||||||||||||||||||||||||||||||||||||||||||||||||||
Sbjct  5112  CCGGGGTACACATTTTGGACTGTGGATCTGCAGGAGCGTTTGTCCTTGGACCTGGACCAG  5171

Query  4561  TACACCCTGGGGAGGCGCTTTTTGGCGCAGTCAGGGGTGTCTACCACACGCTCGCTTAGG  4620
             ||||||||||||||||||||||||||||||||||||||||||||||||||||||||||||
Sbjct  5172  TACACCCTGGGGAGGCGCTTTTTGGCGCAGTCAGGGGTGTCTACCACACGCTCGCTTAGG  5231

Query  4621  GCCCCCACCTCCAGAAAGCGCAAGTCATCAGGGTCGGCCACCAGAAGGACCACCAAAAGA  4680
             ||||||||||||||||||||||||||||||||||||||||||||||||||||||||||||
Sbjct  5232  GCCCCCACCTCCAGAAAGCGCAAGTCATCAGGGTCGGCCACCAGAAGGACCACCAAAAGA  5291

Query  4681  AGGAAATAGTGTGTATGCCATTCTGTTTTTCATATTTAATAAAGACTTTTGAATAAGCAC  4740
             ||||||||||||||||||||||||||||||||||||||||||||||||||||||||||||
Sbjct  5292  AGGAAATAGTGTGTATGCCATTCTGTTTTTCATATTTAATAAAGACTTTTGAATAAGCAC  5351

Query  4741  TGTGTCATTCATGTTCATGCACCGCACCCGGTGCAAGTATGCACCGCCTGGGATATATAC  4800
             ||||||||||||||||||||||||||||||||||||||||||||||||||||||||||||
Sbjct  5352  TGTGTCATTCATGTTCATGCACCGCACCCGGTGCAAGTATGCACCGCCTGGGATATATAC  5411

Query  4801  CGTCCGCGGTGCCATCGCACCTGGTGCTCACTTCGTGCCAGCTGTACCATTGCCATGGAG  4860
             ||||||||||||||||||||||||||||||||||||||||||||||||||||||||||||
Sbjct  5412  CGTCCGCGGTGCCATCGCACCTGGTGCTCACTTCGTGCCAGCTGTACCATTGCCATGGAG  5471

Query  4861  GCAGAGGATTAAGGTAAGTGCTTTTAAATCCTATGtttttttttCTCCATTGTTTAACTG  4920
             ||||||||||||||||||||||||||||||||||||||||||||||||||||||||||||
Sbjct  5472  GCAGAGGATTAAGGTAAGTGCTTTTAAATCCTATGTTTTTTTTTCTCCATTGTTTAACTG  5531

Query  4921  CACAGTTT  4928
             ||||||||
Sbjct  5532  CACAGTTT  5539
```

```
Range 2: 1 to 602

Score:1112 bits(602), Expect:0.0,
Identities:602/602(100%),  Gaps:0/602(0%), Strand: Plus/Plus

Query  6942  CTGTTCTGGTTTAAGGGCAGCATGTCCAATTGTACATTTACACATGGGATTATGCCGCAG  7001
             ||||||||||||||||||||||||||||||||||||||||||||||||||||||||||||
Sbjct  1     CTGTTCTGGTTTAAGGGCAGCATGTCCAATTGTACATTTACACATGGGATTATGCCGCAG  60

Query  7002  TGGCTGCGAGCACAAACCATGCTTAGCCAACAGACTGAGGAGGCGGTCAAATTTGACCTG  7061
             ||||||||||||||||||||||||||||||||||||||||||||||||||||||||||||
Sbjct  61    TGGCTGCGAGCACAAACCATGCTTAGCCAACAGACTGAGGAGGCGGTCAAATTTGACCTG  120

Query  7062  TCAGACATGATACAGTGGGCGCTGGACAATGACATAACTGAGGAGAGCAAATTGGCATAT  7121
             ||||||||||||||||||||||||||||||||||||||||||||||||||||||||||||
Sbjct  121   TCAGACATGATACAGTGGGCGCTGGACAATGACATAACTGAGGAGAGCAAATTGGCATAT  180

Query  7122  GGGTACGCCCTCCTGGCCGAGAGCGACCCCAATGCAGCCGCCTTCCTGTCCTCCAACAAC  7181
             ||||||||||||||||||||||||||||||||||||||||||||||||||||||||||||
Sbjct  181   GGGTACGCCCTCCTGGCCGAGAGCGACCCCAATGCAGCCGCCTTCCTGTCCTCCAACAAC  240

Query  7182  CAGGCAAAACACGTGCGTGACGCGGCCACTATGGTCCGCCATTACAAGCGGGCACAGATG  7241
             ||||||||||||||||||||||||||||||||||||||||||||||||||||||||||||
Sbjct  241   CAGGCAAAACACGTGCGTGACGCGGCCACTATGGTCCGCCATTACAAGCGGGCACAGATG  300

Query  7242  CTGACCATGTCAATGTCCGCATGGGTGCACAGGCGGTGTGAGGCGGTGGAGGAGCAAGGG  7301
             ||||||||||||||||||||||||||||||||||||||||||||||||||||||||||||
Sbjct  301   CTGACCATGTCAATGTCCGCATGGGTGCACAGGCGGTGTGAGGCGGTGGAGGAGCAAGGG  360

Query  7302  GACTGGCGACCAATTATTTATTTTCTGAAATATCAGAATGTGGAAATTGCTGCATTTTTG  7361
             ||||||||||||||||||||||||||||||||||||||||||||||||||||||||||||
Sbjct  361   GACTGGCGACCAATTATTTATTTTCTGAAATATCAGAATGTGGAAATTGCTGCATTTTTG  420

Query  7362  CAGTGCCTCAAACAGTTCTTTAAAGGGGTCCCCAAAAGAAATTGTCTAGTAATCCAGGGA  7421
             ||||||||||||||||||||||||||||||||||||||||||||||||||||||||||||
Sbjct  421   CAGTGCCTCAAACAGTTCTTTAAAGGGGTCCCCAAAAGAAATTGTCTAGTAATCCAGGGA  480

Query  7422  CCACCAAACACAGGCAAGTCCACCTTCTGTATGAGCCTCATCAATTTCCTTGCAGGGAAG  7481
             ||||||||||||||||||||||||||||||||||||||||||||||||||||||||||||
Sbjct  481   CCACCAAACACAGGCAAGTCCACCTTCTGTATGAGCCTCATCAATTTCCTTGCAGGGAAG  540

Query  7482  GTGCTGTCCTTTGTTAATAGCAGGAGCCAGTTCTGGCTTATGCCCCTAGCTGAGACCAAG  7541
             ||||||||||||||||||||||||||||||||||||||||||||||||||||||||||||
Sbjct  541   GTGCTGTCCTTTGTTAATAGCAGGAGCCAGTTCTGGCTTATGCCCCTAGCTGAGACCAAG  600

Query  7542  GT  7543
             ||
Sbjct  601   GT  602

############################################################################


RID: NR7BC4GB114
Job Title:NODE_183_length_7543_cov_4.284892
Program: BLASTN
Query: NODE_183_length_7543_cov_4.284892 ID: lcl|Query_61151(dna) Length: 7543
Subject:NODE_2291_length_1856_cov_4.902192 ID: lcl|Query_61153(dna) Length: 1856

Sequences producing significant alignments:
                                                                   Scientific
Common                     Max    Total Query   E   Per.   Acc.
Description                                                        Name
Name            Taxid      Score  Score cover Value Ident  Len         Accession
```

```
NODE_2291_length_1856_cov_4.902192
0          3422   3422  24%   0.0   99.95  1856       Query_61153

Alignments:

>NODE_2291_length_1856_cov_4.902192
Sequence ID: Query_61153 Length: 1856
Range 1: 1 to 1856

Score:3422 bits(1853), Expect:0.0,
Identities:1855/1856(99%),  Gaps:0/1856(0%), Strand: Plus/Minus

Query  5014  GGGCTTCCGAAACAATGCTGGCACGGAATCTATGGCACAGACCGCCGCGCGTTCTGCCAA  5073
             ||||||||||||||||||||||||||||||||||||||||||||||||||||||||||||
Sbjct  1856  GGGCTTCCGAAACAATGCTGGCACGGAATCTATGGCACAGACCGCCGCGCGTTCTGCCAA  1797

Query  5074  GTTCAGGCGGGCCCAAGCATGACTGTTGGTAGAAAGACCGCCAGCGGTTCTGGTCACCGC  5133
             ||||||||||||||||||||||||||||||||||||||||||||||||||||||||||||
Sbjct  1796  GTTCAGGCGGGCCCAAGCATGACTGTTGGTAGAAAGACCGCCAGCGGTTCTGGTCACCGC  1737

Query  5134  AGGCAGCCAACCGCGCGCGGTCCTCGGAGGCGCCACTTTCAAATTCTGGCGCCAAGCAGA  5193
             ||||||||||||||||||||||||||||||||||||||||||||||||||||||||||||
Sbjct  1736  AGGCAGCCAACCGCGCGCGGTCCTCGGAGGCGCCACTTTCAAATTCTGGCGCCAAGCAGA  1677

Query  5194  AAGAGGGTGTTCCCAACGGTTCTTTCTTGGCACAGGCAGCAACCTCGACCGCGAAAGGTC  5253
             ||||||||||||||||||||||||||||||||||||||||||||||||||||||||||||
Sbjct  1676  AAGAGGGTGTTCCCAACGGTTCTTTCTTGGCACAGGCAGCAACCTCGACCGCGAAAGGTC  1617

Query  5254  TAATAGGGGCGTGATTTTGGACCGTGGTCGGTCTCTGCAAGTTATTCTTTTCATGATGAT  5313
             ||||||||||||||||||||||||||||||||||||||||||||||||||||||||||||
Sbjct  1616  TAATAGGGGCGTGATTTTGGACCGTGGTCGGTCTCTGCAAGTTATTCTTTTCATGATGAT  1557

Query  5314  TGTTGTTAACAATAATATGAAAGATCAGCATGTTCCACGCACCGCCTGCGCCACCCTCAG  5373
             ||||||||||||||||||||||||||||||||||||||||||||||||||||||||||||
Sbjct  1556  TGTTGTTAACAATAATATGAAAGATCAGCATGTTCCACGCACCGCCTGCGCCACCCTCAG  1497

Query  5374  TCCCAGGAGGGGGGATAGACAGTTACACACCAGATGGAGATAGCTGTGCCTGAAAAGCAG  5433
             ||||||||||||||||||||||||||||||||||||||||||||||||||||||||||||
Sbjct  1496  TCCCAGGAGGGGGGATAGACAGTTACACACCAGATGGAGATAGCTGTGCCTGAAAAGCAG  1437

Query  5434  ACAGTAGGCCTGCCCTGTGTCTTTTGTGGACTGGCCATTGGACATTGGGACGCCCACTGG  5493
             ||||||||||||||||||||||||||||||||||||||||||||||||||||||||||||
Sbjct  1436  ACAGTAGGCCTGCCCTGTGTCTTTTGTGGACTGGCCATTGGACATTGGGACGCCCACTGG  1377

Query  5494  TTCGAGCATGGCGTGTACCAGCTGAGCTTCAAGGACGGGAAGCCCCACGCTGCACACCGT  5553
             ||||||||||||||||||||||||||||||||||||||||||||||||||||||||||||
Sbjct  1376  TTCGAGCATGGCGTGTACCAGCTGAGCTTCAAGGACGGGAAGCCCCACGCTGCACACCGT  1317

Query  5554  GATTGCCTTAAGGAGGCGTGCAAGAGAGAGCACGAGAGGCACAGAGACCCCAAGGTGTAC  5613
             ||||||||||||||||||||||||||||||||||||||||||||||||||||||||||||
Sbjct  1316  GATTGCCTTAAGGAGGCGTGCAAGAGAGAGCACGAGAGGCACAGAGACCCCAAGGTGTAC  1257

Query  5614  ACTGGCACTGAGGTGGAGGACACAGTCGGCCTGAAGGTCAAGTGGATCAGCGTGCGCTGC  5673
             ||||||||||||||||||||||||||||||||||||||||||||||||||||||||||||
Sbjct  1256  ACTGGCACTGAGGTGGAGGACACAGTCGGCCTGAAGGTCAAGTGGATCAGCGTGCGCTGC  1197

Query  5674  CACGTCTGCGGCTACCTGCTTACAGAAGACGACAAAGAGTACTGCAAGCTGTGCAGCTTC  5733
             ||||||||||||||||||||||||||||||||||||||||||||||||||||||||||||
Sbjct  1196  CACGTCTGCGGCTACCTGCTTACAGAAGACGACAAAGAGTACTGCAAGCTGTGCAGCTTC  1137

Query  5734  CCCTTCTACCATACACGCGGCCGCTGGCGCACCAGGTGCTACCACTGCAGGGTAGGCGAT  5793
             ||||||||||||||||||||||||||||||||||||||||||||||||||||||||||||
Sbjct  1136  CCCTTCTACCATACACGCGGCCGCTGGCGCACCAGGTGCTACCACTGCAGGGTAGGCGAT  1077
```

```
Query  5794  GAGGAGGATTGAGAGCGACCAAGAAGACAGTGGTGTAGAGTCCGACTTCGAGGAGGAGCA  5853
             ||||||||||||||||||||||||||||||||||||||||||||||||||||||||||||
Sbjct  1076  GAGGAGGATTGAGAGCGACCAAGAAGACAGTGGTGTAGAGTCCGACTTCGAGGAGGAGCA  1017

Query  5854  GGCAGATCGGGAGCCGGAAAGACTGGCTCCATACCGGATTGTGTTGCCCTGTGCGTGCTG  5913
             ||||||||||||||||||||||||||||||||||||||||||||||||||||||||||||
Sbjct  1016  GGCAGATCGGGAGCCGGAAAGACTGGCTCCATACCGGATTGTGTTGCCCTGTGCGTGCTG  957

Query  5914  CGAGTGTCTCCTGCGCATGGTGGTACAGTGCAGCAGTACAGACATCAACGGTCTCAACCG  5973
             ||||||||||||||||||||||||||||||||||||||||||||||||||||||||||||
Sbjct  956   CGAGTGTCTCCTGCGCATGGTGGTACAGTGCAGCAGTACAGACATCAACGGTCTCAACCG  897

Query  5974  CCTGCTCTGCGGTTCCCTGGGCATCCTGTGTCCAACCTGTGCAGTGGAGAGAGGATACCA  6033
             ||||||||||||||||||||||||||||||||||||||||||||||||||||||||||||
Sbjct  896   CCTGCTCTGCGGTTCCCTGGGCATCCTGTGTCCAACCTGTGCAGTGGAGAGAGGATACCA  837

Query  6034  TGGCCGATGAACCAGGTACCCCCGGGGAAGGCACTAGCGGGGCCTGGTTTCTTGCCAGGG  6093
             ||||||||||||||||||||||||||||||||||||||||||||||||||||||||||||
Sbjct  836   TGGCCGATGAACCAGGTACCCCCGGGGAAGGCACTAGCGGGGCCTGGTTTCTTGCCAGGG  777

Query  6094  AAGCGGTATGTAGTGGGTCAGATAGCGACGAAGAGGAGGCGGCTGATGTGTATGAAGGCC  6153
             ||||||||||||||||||||||||||||||||||||||||||||||||||||||||||||
Sbjct  776   AAGCGGTATGTAGTGGGTCAGATAGCGACGAAGAGGAGGCGGCTGATGTGTATGAAGGCC  717

Query  6154  CAGACCTAGACTTTGTAGACAATGCCTCCTATCACCAGGGAAATTCCCTGTACCTCCTCC  6213
             ||||||||||||||||||||||||||||||||||||||||||||||||||||||||||||
Sbjct  716   CAGACCTAGACTTTGTAGACAATGCCTCCTATCACCAGGGAAATTCCCTGTACCTCCTCC  657

Query  6214  AACAGCAAGAAGCGGAGGAGGACCGGCTGCATGTGCAGCTGCTAAAACGAAAATATGTAG  6273
             ||||||||||||||||||||||||||||||||||||||||||||||||||||||||||||
Sbjct  656   AACAGCAAGAAGCGGAGGAGGACCGGCTGCATGTGCAGCTGCTAAAACGAAAATATGTAG  597

Query  6274  AGAGCCCTAAACAGCGATTATGCCTGGCGCTCAGCCCCAGGCTGCAGGCTATTCAAATTT  6333
             ||||||||||||||||||||||||||||||||||||||||||||||||||||||||||||
Sbjct  596   AGAGCCCTAAACAGCGATTATGCCTGGCGCTCAGCCCCAGGCTGCAGGCTATTCAAATTT  537

Query  6334  CCCCCAATAAACAGCCTGTGAAAAGGCGCCTGTTTCAGCATCAGGACCAGGACAGCGGTC  6393
             ||||||||||||||||||||||||||||||||||||||||||||||||||||||||||||
Sbjct  536   CCCCCAATAAACAGCCTGTGAAAAGGCGCCTGTTTCAGCATCAGGACCAGGACAGCGGTC  477

Query  6394  TGGACCTGTCTCTGCAAAATGAAACTGCTAGTAATGACCAGGACGGGACCCACATTCAGG  6453
             ||||||||||||||||||||||||||||||||||||||||||||||||||||||||||||
Sbjct  476   TGGACCTGTCTCTGCAAAATGAAACTGCTAGTAATGACCAGGACGGGACCCACATTCAGG  417

Query  6454  TAGACCCATGCGAAACTGTGGGGGACAGGAATGGAAATGGCGAAgggggggCGCGAGCCG  6513
             ||||||||||||||||||||||||||||||||||||||||||||||||||||||||||||
Sbjct  416   TAGACCCATGCGAAACTGTGGGGGACAGGAATGGAAATGGCGAAGGGGGGGCGCGAGCCG  357

Query  6514  GGGAGACCCCCTGGCAGCCAAGTAAGGCCGCCAGTGGGACAGAGGCACAGCCGCATCTGG  6573
             ||||||||||||||||||||||||||||||||||||||||||||||||||||||||||||
Sbjct  356   GGGAGACCCCCTGGCAGCCAAGTAAGGCCGCCAGTGGGACAGAGGCACAGCCGCATCTGG  297

Query  6574  ACGTACTCAGAGCCAGTAATCGGAGGGCGGCCATGTTGGCGCGCTTTAAAGAAGGCTTCG  6633
             ||||||||||||||||||||||||||||||||||||||| ||||||||||||||||||||
Sbjct  296   ACGTACTCAGAGCCAGTAATCGGAGGGCGGCCATGTTGGTGCGCTTTAAAGAAGGCTTCG  237

Query  6634  GTGTCAGCTTTTCTGAGCTCACGCGCAGCTTTAAAAGCGACCGCTCCTGTGTGGGGGACT  6693
             ||||||||||||||||||||||||||||||||||||||||||||||||||||||||||||
Sbjct  236   GTGTCAGCTTTTCTGAGCTCACGCGCAGCTTTAAAAGCGACCGCTCCTGTGTGGGGGACT  177

Query  6694  GGGTCGTGCTGGCGTTTGGCGTCCGAGAACAGTTTGCCGAAACCGCCAAAGAGCAGCTGA  6753
             ||||||||||||||||||||||||||||||||||||||||||||||||||||||||||||
Sbjct  176   GGGTCGTGCTGGCGTTTGGCGTCCGAGAACAGTTTGCCGAAACCGCCAAAGAGCAGCTGA  117
```

```
Query  6754  AGGGACACTGTGGATACGTGCAGTTCACGTATCGACCGGACGCTAGGGGTGCGCTTACAC  6813
             ||||||||||||||||||||||||||||||||||||||||||||||||||||||||||||
Sbjct  116   AGGGACACTGTGGATACGTGCAGTTCACGTATCGACCGGACGCTAGGGGTGCGCTTACAC  57

Query  6814  TAGCGCTGCTGTCATTTACCTGTCAAAAGAATAGGGACACTGTACGTAACCTGATG  6869
             ||||||||||||||||||||||||||||||||||||||||||||||||||||||||
Sbjct  56    TAGCGCTGCTGTCATTTACCTGTCAAAAGAATAGGGACACTGTACGTAACCTGATG  1
```
